# Unique circulating miRNAome signatures captured with distinct molecular states in Lynch syndrome and sporadic colorectal cancer

**DOI:** 10.1002/1878-0261.70338

**Published:** 2026-10-07

**Authors:** Ramadhani Salum Chambuso, Md Shohel Ul Islam, Rosa M Xicola, Raj Ramesar, Xavier Llor, Sinclair Steele

**Affiliations:** ^1^ Department of Pathological Sciences, College of Medicine Ajman University UAE; ^2^ Department of Global Health and Population Harvard T.H. Chan School of Public Health Boston MA USA; ^3^ Department of Genetic Engineering and Biotechnology, Faculty of Biological Science and Technology Jashore University of Science and Technology Bangladesh; ^4^ Section of Digestive Diseases, Department of Internal Medicine Yale School of Medicine New Haven CT USA; ^5^ Division of Human Genetics, Department of Pathology University of Cape Town South Africa

**Keywords:** age‐stratified transcriptomics, circulating microRNAs, colorectal cancer risk profiling, Lynch syndrome biomarkers, miRNA‐mRNA regulatory networks, orthogonal validation

## Abstract

Hereditary nonpolyposis colorectal cancer (HNPCC), usually termed Lynch syndrome (LS), is the most prevalent hereditary colorectal cancer syndrome, yet molecular biomarkers to differentiate nonmalignant from malignant LS cases remain limited. We performed a secondary analysis of the circulating miRNAome in 155 individuals, including healthy controls (CTRL), nonmalignant LS (H‐LS), cancer‐associated LS (C‐LS), and sporadic colorectal cancer (CRC). We observed that LS variant heterozygotes, especially those without overt cancer, exhibit a transitional miRNAomic architecture distinct from both healthy controls and CRC patients in different age groups. Age‐stratified differential expression and miRNA‐mRNA network analyses revealed distinct regulatory hubs across groups. Group‐specific miRNAs targeted oncogenic, immune, and DNA repair pathways. The analysis suggested that nonmalignant LS variant heterozygotes occupy a circulating miRNAome state distinct from both healthy controls and overt CRC, whereas C‐LS and sporadic CRC shared selected malignant‐associated miRNA features. To assess external concordance, we performed orthogonal validation of a prespecified six‐miRNA panel in the independent GSE30454 primary colorectal tissue cohort, comprising normal, LS, sporadic MSI, and MSS samples. The composite panel preserved disease‐axis structure across the external cohort, with strongest discrimination in biologically relevant mismatch repair‐related contrasts. These findings suggest candidate circulating miRNA molecular states across LS and sporadic colorectal cancer.

## Graphical abstract

Circulating microRNA profiles distinguish nonmalignant Lynch syndrome from cancer‐associated Lynch syndrome, sporadic colorectal cancer, and healthy controls. Age‐stratified analysis reveals disease‐specific regulatory states, and a six‐miRNA panel shows concordant separation in an independent colorectal tissue cohort. These findings support circulating miRNA signatures as candidates for molecular surveillance and risk stratification in Lynch syndrome. Artwork source: Created as original vector artwork using Python 3 and ReportLab; graphical assets were used.

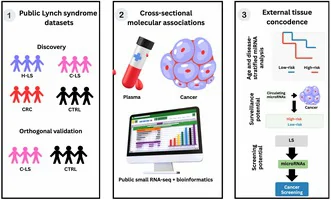

**Abbreviations**
AGE‐RAGE: advanced glycation end products‐receptor for advanced glycation end products
ANOSIM: analysis of similarities
AUC: area under the receiver operating characteristic curve
BioCarta: BioCarta pathway database
BP: biological process
CC: cellular component
CI: confidence interval
C‐LS: cancer‐associated Lynch syndrome
CRC: sporadic colorectal cancer
CTRL: healthy control
DE: differential expression
DESeq2: Differential Expression Sequencing 2 R package
EPCAM: epithelial cell adhesion molecule
FDR: false discovery rate
GEO: Gene Expression Omnibus
GO: Gene Ontology
H‐LS: nonmalignant Lynch syndrome
HNPCC: hereditary nonpolyposis colorectal cancer
ImmuneSigDB: Immunologic Signature Gene Set Database
IQR: interquartile range
KEGG: Kyoto Encyclopedia of Genes and Genomes
LS: Lynch syndrome
MAPK: mitogen‐activated protein kinase
MF: molecular function
microRNA: miRNA/miR
miRTarBase: experimentally validated miRNA‐target interaction database
MLH1: MutL homolog 1
MMR: mismatch repair
mRNA: messenger RNA
MSH2: MutS homolog 2
MSH6: MutS homolog 6
MSI: microsatellite instability
MSS: microsatellite stable
PCA: principal component analysis
PCoA: principal coordinates analysis
PERMANOVA: permutational multivariate analysis of variance
PERMDISP: permutational analysis of multivariate dispersions
PID: Pathway Interaction Database
PMS2: postmeiotic segregation increased 2
qPCR: quantitative polymerase chain reaction
ROC: receiver operating characteristic
SRA: Sequence Read Archive
TMM: trimmed mean of M‐values
UMAP: uniform manifold approximation and projection
VST: variance‐stabilizing transformation
WGCNA: weighted gene co‐expression network analysis

## Introduction

Lynch syndrome (LS) is the most common hereditary colorectal cancer syndrome, responsible for 2–4% of all sporadic colorectal cancer (CRC) cases worldwide [1]. LS arises from germline mutations in DNA mismatch repair (MMR) genes, resulting in microsatellite instability and a markedly elevated lifetime risk of colorectal, endometrial, and other epithelial malignancies [1, 2]. While genetic testing has improved early identification, it remains clinically difficult to distinguish nonmalignant LS variant heterozygotes from those progressing toward malignancy at a molecular level [2, 3, 4, 5]. Current surveillance strategies rely primarily on colonoscopy and family history, offering limited insight into the real‐time molecular changes driving carcinogenic transformation. There remains a critical need for dynamic, noninvasive immunogenomic and transcriptome‐based biomarkers capable of capturing early temporal molecular changes according to aging in LS, despite the fact that age is a significant risk factor for LS [6, 7, 8, 9, 10].

We have previously published a study on the identification of novel immunogenomic biomarkers in LS and discussed about the use of neoantigens‐triggered immune responses as novel blood‐based biomarkers using immunogenomic markers or gene expression profiles for LS cancer initiation [11]. However, microRNAs (miRNAs) are small, noncoding RNAs that regulate gene expression post‐transcriptionally and are increasingly recognized as master regulators of cancer‐related processes, including immune signaling, DNA repair, and apoptosis [12, 13].

Circulating miRNAs are highly stable in plasma, readily detectable and minimally invasive to measure, making them attractive candidates for blood‐based biomarkers [14, 15]. However, little is known about how the circulating miRNAome differs with age across healthy controls, nonmalignant LS variant heterozygotes and CRC [15, 16, 17, 18, 19, 20]. Furthermore, aging, a key modulator of immune function, inflammation and genomic stability, likely alters miRNA‐mediated regulation, yet its role in shaping the miRNAome within these at‐risk populations remains poorly understood [20, 21, 22]. Recent network‐based approaches have demonstrated that miRNA‐mRNA interaction mapping can uncover disease‐specific regulatory architectures and inform patient stratification [23, 24, 25]. While such strategies have been applied to various solid tumors, little effort has been made to profile the age‐stratified circulating miRNAome landscape in LS [5]. Addressing this gap may yield critical insights into early immunogenomic rewiring, enabling precision surveillance and risk prediction in LS variant heterozygotes [1, 11, 26].

In this study, we performed a secondary bioinformatic analysis of publicly available circulating miRNA data from 155 individuals across four clinical groups: healthy controls, nonmalignant LS (H‐LS), cancer‐associated LS (C‐LS), and CRC. We used age‐stratified differential expression, dimensionality reduction, enrichment, and miRNA‐mRNA network analyses to identify unique candidate molecular‐state signatures associated with LS and sporadic CRC.

## Materials and methods

### Ethics statement

As the study used publicly available de‐identified data, no additional ethical approval was required.

### Data source and study design

This was a retrospective study design as a secondary bioinformatic analysis of publicly available miRNA datasets which leveraged publicly available transcriptomic data from the Gene Expression Omnibus (GEO) under accession number GSE198834 for the discovery cohort. The dataset includes small RNA‐sequencing profiles of circulating microRNAs (c‐miRs) derived from peripheral blood samples of healthy control individuals (CTRL), LS variant heterozygotes with or without cancer (C‐LS and H‐LS, respectively), and CRC patients. External orthogonal validation was performed using a publicly available GSE30454 colorectal primary‐tissue miRNA cohort, which included normal tissue, LS, sporadic MSI, and MSS classes to assess orthogonal concordance of a focused miRNA panel in an independent primary colorectal tissue cohort, but this validation was not interpreted as definitive clinical validation of a plasma‐based assay.

### Sample processing and data acquisition

The GSE198834 dataset was accessed through the Gene Expression Omnibus (GEO), from which the processed miRNA count matrix and corresponding sample metadata were obtained for downstream analysis. Metadata, including age, sex, and disease status, were extracted from the associated GEO records and cross‐checked against the original sample annotations. No patient recontact was required because all data were publicly available and de‐identified. The curated demographic and clinical metadata are provided in Table S1. The secondary‐analysis framework followed established integrated expression and network analysis approaches [27, 28, 29].

### Clinical cohort description

All peripheral plasma samples included in this study were derived from the publicly available dataset (GSE198834) and comprised individuals categorized into four clinically distinct groups:

- Healthy controls (CTRL) *n* = 37
- LS without cancer (H‐LS) *n* = 83
- LS with cancer (C‐LS) *n* = 11
- Sporadic CRC *n* = 24.

Subjects were stratified into three age categories: ≤ 40 years (young), 41–60 years (middle), and > 60 years (older). Classification of LS was based on germline pathogenic variants in mismatch repair genes (*MLH1, MSH2, MSH6, PMS2*, *and EPCAM*) and clinical diagnosis was confirmed in the original dataset through pedigree analysis, genetic counseling, and molecular testing.

### Sample collection and processing

Detailed sample processing protocols were described in the original GSE198834 study. Plasma was collected, processed, and stored under standardized conditions minimizing pre‐analytical variation [16].

### RNA isolation from plasma samples

The processed miRNA count matrix and associated sample metadata for the GSE198834 dataset were obtained from the GEO for downstream analysis. The dataset included total RNA and small RNAs extracted from plasma samples according to the standardized protocol described by the original data generators. Plasma samples were processed to ensure the recovery of both long and small RNA species, maximizing the capture of miRNAs and other noncoding RNAs relevant to our study [30]. RNA isolation was performed using a robust protocol specifically designed for biofluid samples, which inherently contain lower RNA quantities and were prone to protein contamination. The isolation method employed a combination of organic extraction and column‐based purification to ensure high quality [31]. The unique molecular identifiers (UMIs) were included in the original library preparation [16]. While UMIs are useful in eliminating PCR duplicates especially in low‐input RNA‐seq studies, existing studies have shown that standard small RNA protocols when combined with appropriate filtering thresholds and statistical modeling remain robust for population‐level miRNA differential expression analyses [32, 33, 34]. Small RNA sequencing, preprocessing, and direct assessment of read quality (e.g., with FastQC) was performed according to the details in the original study [16].

### Bioinformatics and differential expression analysis

Normalized expression matrices were batch‐corrected using ComBat from the sva package. Differential expression (DE) was analyzed using limma‐voom and edgeR with FDR < 0.05 considered significant. Volcano plots and heatmaps were generated to visualize differentially expressed miRNAs as explained in the original study [35]. Briefly, preprocessed normalized miRNA expression matrices were first evaluated for batch effects using principal component analysis (PCA) and subsequently corrected using the ComBat function from the sva package (RRID:SCR_000099, https://scicrunch.org/resolver/RRID:SCR_000099) to adjust for known covariates such as sequencing batch and RNA extraction date. Sample quality control was performed using total read distribution, boxplots and hierarchical clustering to exclude outliers. For DE analysis, we employed a multi‐method pipeline combining both limma‐voom (RRID:SCR_010943, https://scicrunch.org/scicrunch/resolver/RRID:SCR_010943) and edgeR (RRID:SCR_012802, https://scicrunch.org/resolver/SCR_012802) to enhance robustness and consistency. Raw counts were filtered using the filterByExpr function in edgeR, excluding miRNAs with less than 1 count per million in ≥ 70% of the samples to reduce noise and improve statistical power. Normalization was performed using the trimmed mean of M‐values (TMM) method and log2‐transformed expression values were calculated using the voom function with precision weights to account for mean–variance relationships in RNA‐Seq data [36, 37]. Supporting count, normalized expression, regression and disease‐contrast outputs are provided in Tables S2–S13.

### miRNA‐mRNA target network analysis

The most upregulated miRNAs were selected for miRNA‐mRNA target network analysis. These miRNAs were submitted to the https://www.mirnet.ca/ to identify their target mRNAs. The resulting network file was visualized using Cytoscape (version 3.10.3), shown in Fig. S1. Briefly, to investigate the regulatory landscape and downstream impact of circulating miRNAs, we constructed integrative miRNA‐mRNA target networks leveraging both experimentally validated and computationally predicted interactions [38, 39]. Predicted miRNA targets were retrieved from TargetScan Human 8.0 (RRID:SCR_010845, https://www.targetscan.org/vert_80/), which provides context++ scores for conserved and poorly conserved miRNA binding sites. Experimentally validated miRNA‐mRNA interactions were extracted from miRTarBase v9.0 (RRID:SCR_017400, https://rrid.site/data/record/nlx_144509‐1/SCR_017400/resolver?q=%2A&l=&facet[]=Keywords%3Agenetic&facet[]=Resource%20Type%3Adata%20analysis%20software&sort=desc&column=Record%20Last%20Update&sort=desc&i=rrid:scr_017400), prioritizing targets supported by strong experimental evidence such as reporter assays, western blot, and qPCR. Following target retrieval, a merged interaction list was curated, ensuring that for each miRNA, only high‐confidence targets (conserved sites in TargetScan or validated targets in miRTarBase) were retained. Redundant interactions and low‐confidence predictions were filtered out to reduce noise in the downstream network modeling [40].

To visualize and analyze the global architecture of miRNA‐target interactions, we employed Cytoscape, (RRID:SCR_003032, https://cytoscape.org/cy3.html) to construct directed, bipartite networks where nodes represent miRNAs or mRNA targets and edges represent regulatory interactions. Network topology was quantified using standard centrality metrics: degree centrality (number of connections per node) identified high‐connectivity miRNAs, betweenness centrality captured nodes bridging multiple subnetworks and closeness centrality assessed how efficiently a node communicates with all others. Regulatory hubs were defined as miRNAs ranking in the top 5% across at least two centrality metrics [41, 42].

To uncover modules of miRNAs with shared expression patterns, Weighted Gene Co‐expression Network Analysis (WGCNA) (RRID:SCR_003302, https://scicrunch.org/resolver/RRID:SCR_003302) was applied to variance‐stabilizing transformation (VST)‐normalized expression data obtained from DESeq2 [43]. A soft‐thresholding power was chosen to approximate scale‐free topology (*R* ^2^ ≥ 0.85) and unsigned co‐expression networks were constructed to identify biologically meaningful correlations regardless of direction. Dynamic tree cutting was used to detect modules with a minimum module size of 10 miRNAs. Each module was summarized by its eigengene (first principal component of expression) and eigengenes were then correlated with clinical traits including: Cancer status (CTRL, H‐LS, C‐LS, CRC), immune cell infiltration scores (derived from MCP‐counter, RRID:SCR_017296, https://dknet.org/data/record/nlx_144509‐1/RRID:SCR_017296/resolver) and the top modules with the strongest correlation to disease phenotype (Pearson |r| > 0.6, FDR < 0.05) were further explored for functional enrichment and hub miRNA membership overlap with the miRNA‐mRNA regulatory network [44, 45]. Module membership and trait‐correlation outputs are provided in Tables S14–S17.

### miRNA pathway and enrichment analysis

Pathway and functional enrichment analyses, including Gene Ontology (GO), KEGG, BioCarta, Reactome, PID, and ImmuneSigDB, were performed using the clusterProfiler, org.Hs.eg.db, msigdbr, and ReactomePA packages in R (version 4.2.1). To explore the biological relevance of differentially expressed miRNAs, we performed pathway and enrichment analysis using multiple bioinformatics tools and publicly available databases [46, 47]. The primary aim was to identify key signaling pathways and biological processes modulated by the miRNAs identified in our study.

### Data preparation and preprocessing

Initially, the predicted and validated target genes of the DE miRNAs were retrieved from the miRTarBase database (release 9.0), which provides comprehensive experimentally validated miRNA‐target interactions [48]. This database was chosen due to its robust curation of miRNA‐gene relationships, including evidence from high‐throughput sequencing, reporter assays, and other functional validation studies.

### Heatmap

Heatmaps were generated with pheatmap, tidyr, dplyr, and tibble in R version 4.2.1. Normalized expression matrices and heatmap input data are provided in Tables S2 and S3; the corresponding visualizations are shown in Figs S2–S6.

### UMAP and PCoA analysis

UMAP and principal coordinates analysis (PCoA) were performed with the UMAP, vegan, and ape packages. PERMANOVA was used to test centroid separation. Differential expression diagnostic plots and covariate‐contrast volcano plots are shown in Figs S7–S13.

### Circos analysis

Highly expressed miRNAs from each age group were selected and visualized using Tbtools (version 2.225).

### Enrichment analysis tools and parameters

We utilized two R packages, clusterProfiler (v4.6.0) and enrichR, to perform enrichment analysis and pathway annotation. The clusterProfiler package was chosen for its ability to perform statistical analysis and visualization of functional profiles, including Gene Ontology (GO) and KEGG pathway enrichment. Additionally, enrichR was employed for accessing multiple databases simultaneously, including KEGG, Reactome, and miEAA, which provides miRNA‐specific enrichment analysis [49].

### Functional enrichment analysis

Target gene sets derived from the predicted miRNA interactions were subjected to pathway enrichment analysis [50, 51, 52]. The following steps were undertaken:

- GO enrichment:
  - The GO analysis focused on three domains: Biological Processes (BP), Cellular Components (CC), and Molecular Functions (MF).
  - Enrichment analysis was conducted using the clusterProfiler package, employing the enrichGO function to categorize genes into relevant biological processes and molecular functions.
  - Significant GO terms were selected based on a false discovery rate (FDR) cut‐off of < 0.05 using the Benjamini–Hochberg correction method.
- KEGG pathway analysis:
  - KEGG pathway annotations were obtained using the enrichKEGG function within clusterProfiler.
  - This analysis identified canonical pathways significantly associated with the miRNA‐target gene sets.
  - Pathways were ranked based on adjusted *P* values, with only those having FDR < 0.05 considered significant.
- Reactome pathway analysis:
  - We employed enrichR to perform Reactome pathway enrichment.
  - The analysis targeted pathway‐specific interactions that could reveal mechanistic insights into miRNA regulatory roles.
- miEAA enrichment analysis:
  - To specifically investigate miRNA‐related pathways, the miEAA tool was used.
  - The miEAA enrichment analysis included databases specifically curated for miRNA‐target interactions, offering additional specificity to the pathway mapping process.

### Data visualization and interpretation

Enrichment results from each analysis were visualized as follows:

- Dot plots: Displaying the most significantly enriched pathways with their respective FDR values.
- Bar plots: Ranking pathways according to their enrichment scores and statistical significance.
- Network plots: Depicting the relationships between miRNAs and their significantly enriched pathways.

### Validation of enrichment findings

To ensure the robustness of our findings, we cross‐validated the enriched pathways using multiple databases, including the Molecular Signatures Database (MSigDB) [53]. Comparative analysis with experimentally validated pathways was conducted to confirm the biological relevance of the results.

### Outcome and interpretation

The pathway analysis revealed significant enrichment in immune response pathways, oncogenic signaling networks, and metabolic regulation, highlighting potential mechanisms by which miRNA dysregulation influences colorectal cancer progression. The integration of multiple pathway databases ensured comprehensive coverage of potential biological processes affected by the differentially expressed miRNAs [54, 55].

### Immune infiltration and immunoregulatory signature analysis

Immune cell infiltration signatures were estimated using the MCP‐counter algorithm on matched gene expression data. For immune checkpoint analysis, correlation matrices were computed between hub c‐miRs from modules and expression levels of canonical checkpoint genes (PDCD1, CTLA4, LAG3, TIGIT) [56].

### Visualization tools and software

Figures were generated with ggplot2, GraphPad Prism 9, and Seaborn; Cytoscape version was used for network visualization and ComplexHeatmap for heatmaps. DESeq2, dplyr, ggplot2, ggrepel, and apeglm were used in R version 4.2.1. Disease‐contrast volcano plots are shown in Figs S14–S22.

### External orthogonal validation using GSE30454 primary colorectal tissue

External orthogonal validation was performed using the publicly available GSE30454 colorectal primary‐tissue miRNA cohort, which included normal tissue, LS, sporadic MSI, and MSS classes. Probe‐level expression values corresponding to the six prespecified miRNAs: miR‐486‐5p, miR‐183, miR‐130b, miR‐598, miR‐99a, and miR‐106b were extracted from the series matrix and matched to published sample annotations. This panel was selected before external testing because the miRNAs were biologically central to the discovery analysis, technically represented in the validation cohort, and supported by microarray and/or qPCR evidence in the source study.

For multiclass visualization, row‐wise z‐scores were used for heatmap generation, and unsupervised low‐dimensional projection was used to visualize class‐level structure and miRNA loading directions. A composite six‐miRNA validation score was calculated from standardized expression values and compared across classes using nonparametric group testing with pairwise comparisons. The primary ROC‐based validation contrast was LS versus sporadic MSI, selected because both groups are mismatch repair‐deficient and therefore represent a more stringent specificity test than tumor‐versus‐normal discrimination. Secondary contrasts were all tumors versus normal and MSI‐related versus MSS. Classification performance was summarized using ROC curves, AUC, accuracy, recall, precision, specificity, F1 score, and Cohen's kappa. Standardized effect sizes were estimated for key contrasts, and a published‐support matrix was curated from the source study's microarray and qPCR supplementary results to distinguish new reanalysis‐based external support from previously reported evidence classes [57, 58, 59].

### Statistical analysis

Because the cancer‐associated LS subgroup was small, subgroup‐level analyses involving C‐LS, age‐by‐disease strata and MMR gene‐specific categories were treated as exploratory. These analyses were retained to describe candidate biological patterns in a rare disease dataset, but they were not interpreted as definitive candidate disease‐associated patterns. Where appropriate, nonparametric tests and FDR‐aware interpretation were used, and effect patterns were prioritized over claims of clinical classification. Enrichment terms with an FDR of < 0.05 were retained for further analysis. The statistical significance was evaluated using the hypergeometric test implemented in clusterProfiler, followed by the Benjamini–Hochberg correction to minimize false positives [60]. RStudio was used for correlation and regression analyses. Identified modules of c‐miRs that were co‐expressed were correlated with age, CRC status, or LS. An ensemble model was built using regularized logistic regression, random forest and support vector machine (SVM) classifiers. Input features were expression values of miRs from significantly enriched modules. Performance was assessed using 10‐fold cross‐validation. Models were implemented using the caret and mlr3 packages [61]. All statistical analyses were conducted using R v4.2.1. Kruskal–Wallis and Dunn's post hoc tests were used for group comparisons. Chi‐square and Fisher's exact tests were applied for categorical data. UMAP and PCoA were performed using UMAP and vegan packages. PERMANOVA was used to assess cluster significance [62]. Statistical significance was set at *P* < 0.05 unless otherwise indicated. Age association, cancer association and regression outputs are provided in Tables S18–S20. Disease‐contrast differential expression results and miRNA‐target interaction outputs are provided in Tables S21–S32. For age‐by‐disease comparisons, ordinary least‐squares models included continuous age, disease status (CRC versus CTRL) and their interaction; two‐sided *P* values for the age × disease‐status interaction were used to compare slopes, and R‐squared reports the full model coefficient of determination.

## Results

### Age‐stratified miRNA expression reveals distinct signatures across colorectal cancer subtypes

We first examined the expression dynamics of 12 top‐ranked miRNAs with significant FDR (*q*‐values) across three age strata (≤ 40 or young, 41–60 or middle, and > 60 years old or older), which revealed consistent age‐dependent transcriptional patterns (Fig. 1A). Several miRNAs demonstrated a decrease in expression with older age, while others exhibited peak expression in midlife, suggesting nonlinear regulation aligned with transitional physiological states [63, 64, 65, 66, 67, 68]. We next evaluated whether miRNA expression profiles varied by disease groups (treatment) H‐LS, CRC, C‐LS, and CTRL (Fig. 1B). General observation showed different microRNAs' expression according to treatment groups (H‐LS compared to C‐LS, CRC, and CTRL), suggesting that their altered expression in H‐LS may reflect the interplay between germline DNA repair deficiency and immune escape pathways and distinct etiological mechanisms underpinning tumor initiation [69, 70, 71, 72, 73]. To further explore age‐specific miRNA dysregulation, we performed an overlap analysis of significantly altered miRNAs across the three age groups (≤ 40, 41–60, > 60 years; Fig. 1C). The majority of miRNAs were unique to each age category, with 42 miRNAs (26%) exclusive to the ≤ 40 group, 35 (22%) to the 41–60 group, and 55 (34%) to the > 60 group. Only one miRNA (0.62%) was common across all age strata. This finding suggests that miRNA regulatory programs undergo distinct shifts at different life stages, rather than progressing in a linear age continuum [21, 74, 75]. Such divergence reinforces the biological necessity for age‐stratified models when interpreting miRNA‐based diagnostics or therapeutic targets [74]. (Figs S3–S6).

**Fig. 1:**
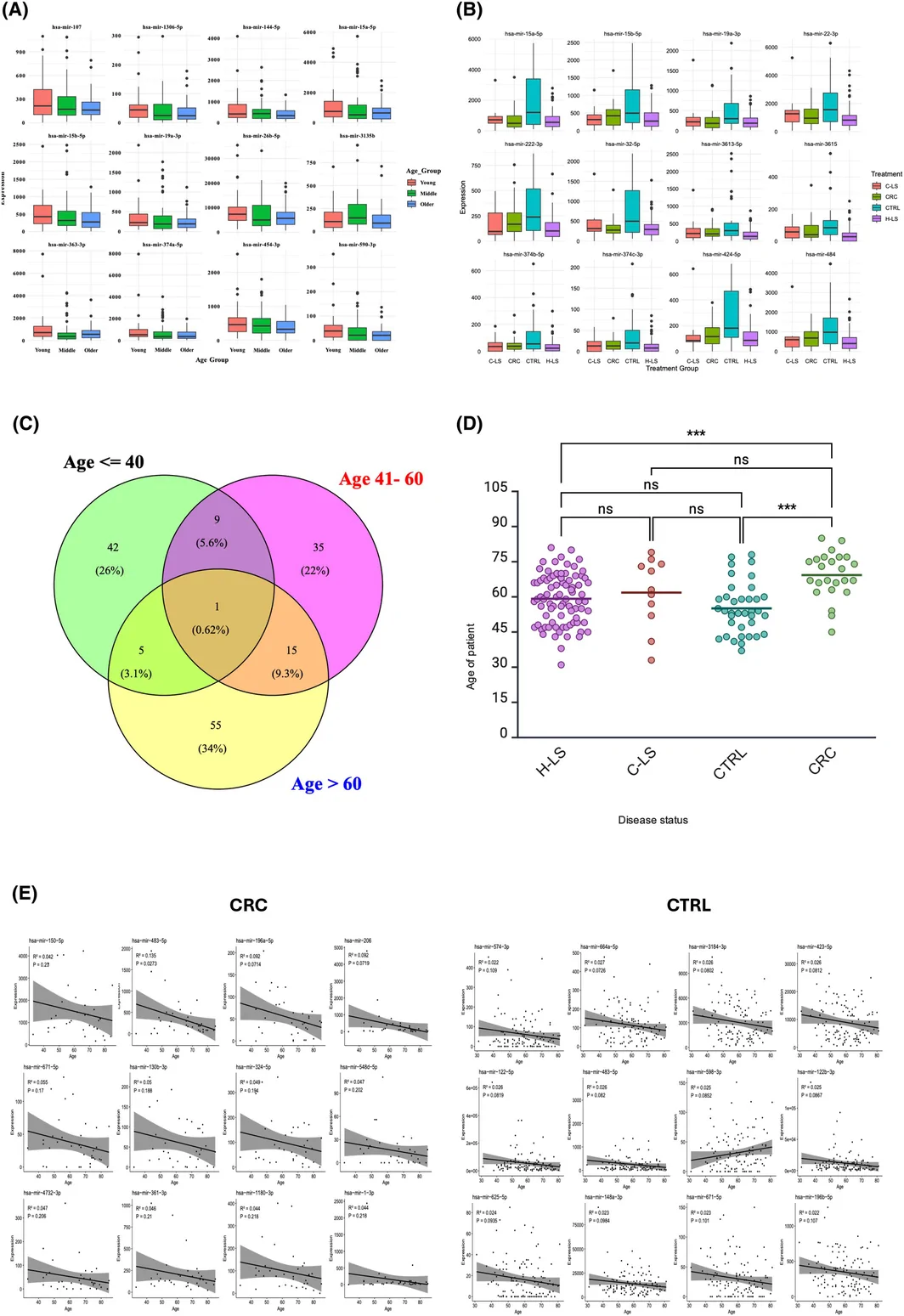
Age‐ and disease‐associated circulating miRNA patterns. (A) Normalized expression of 12 top‐ranked miRNAs across participants aged ≤ 40 years (young), 41–60 years (middle), and > 60 years (older). (B) Normalized expression of 12 miRNAs across C‐LS, CRC, CTRL, and H‐LS clinical groups. In A and B, the center line is the median, the box spans the interquartile range (IQR), whiskers extend to 1.5× IQR, and points beyond the whiskers are outliers. (C) Venn diagram showing the number and percentage of significantly altered miRNAs unique to or shared among the three age groups. (D) Age distribution by clinical group; points represent participants, and the horizontal line represents the median. Pairwise differences were assessed with two‐sided Mann–Whitney U‐tests; ***P < 0.001 and ns, *P* ≥ 0.05. (E) Expression‐versus‐age scatterplots for selected miRNAs in CRC and CTRL; lines are ordinary least‐squares fits, and gray bands are 95% confidence intervals. C‐LS, cancer‐associated Lynch syndrome; CRC, sporadic colorectal cancer; CTRL, healthy control; H‐LS, nonmalignant Lynch syndrome; hsa, Homo sapiens; miRNA/miR, microRNA.

To assess age distribution across the cohort, we plotted patient ages for H‐LS, C‐LS, CTRL, and CRC groups (Fig. 1D). Patients with CRC were significantly older than both the H‐LS and CTRL groups (*P* < 0.001, Mann–Whitney U‐test), consistent with the typical late‐onset nature of CRC [76, 77]. In contrast, no significant age differences were observed among the H‐LS, C‐LS, and CTRL groups, indicating demographic comparability among these cohorts. The median age of H‐LS patients (~ 60 years) deepens the necessity for molecular surveillance strategies beyond age‐based screening thresholds.

To identify miRNAs most tightly linked to age, we examined the expression trends of 12 candidate miRNAs across the full age spectrum for CRC versus CTRL disease status using linear regression (Fig. 1E). All displayed miRNAs except miRNA 598‐3p, exhibited negative correlations with increasing age, as reflected by consistently downward correlation slopes, reinforcing the overarching pattern of miRNA downregulation with aging [20, 78]. These results define a robust, age‐stratified miRNA signature landscape across different disease statuses. The findings support the hypothesis that age is not merely a confounding factor but a primary axis of miRNA regulation, intersecting with inherited and sporadic oncogenic pathways [20, 78, 79].

### Integrated miRNA expression patterns reveal clinically and age‐distinct molecular subtypes

We next explored the global architecture of miRNA expression across the cohort to uncover latent molecular patterns associated with clinical phenotype and age. Unsupervised hierarchical clustering of all miRNAs uncovered a layered expression landscape in different groups (Fig. 2A). When we zoomed in on the most differentially expressed miRNAs (Fig. 2B), these distinctions became sharper, with a small number of miRNAs accounting for most of the group‐level divergence.

**Fig. 2:**
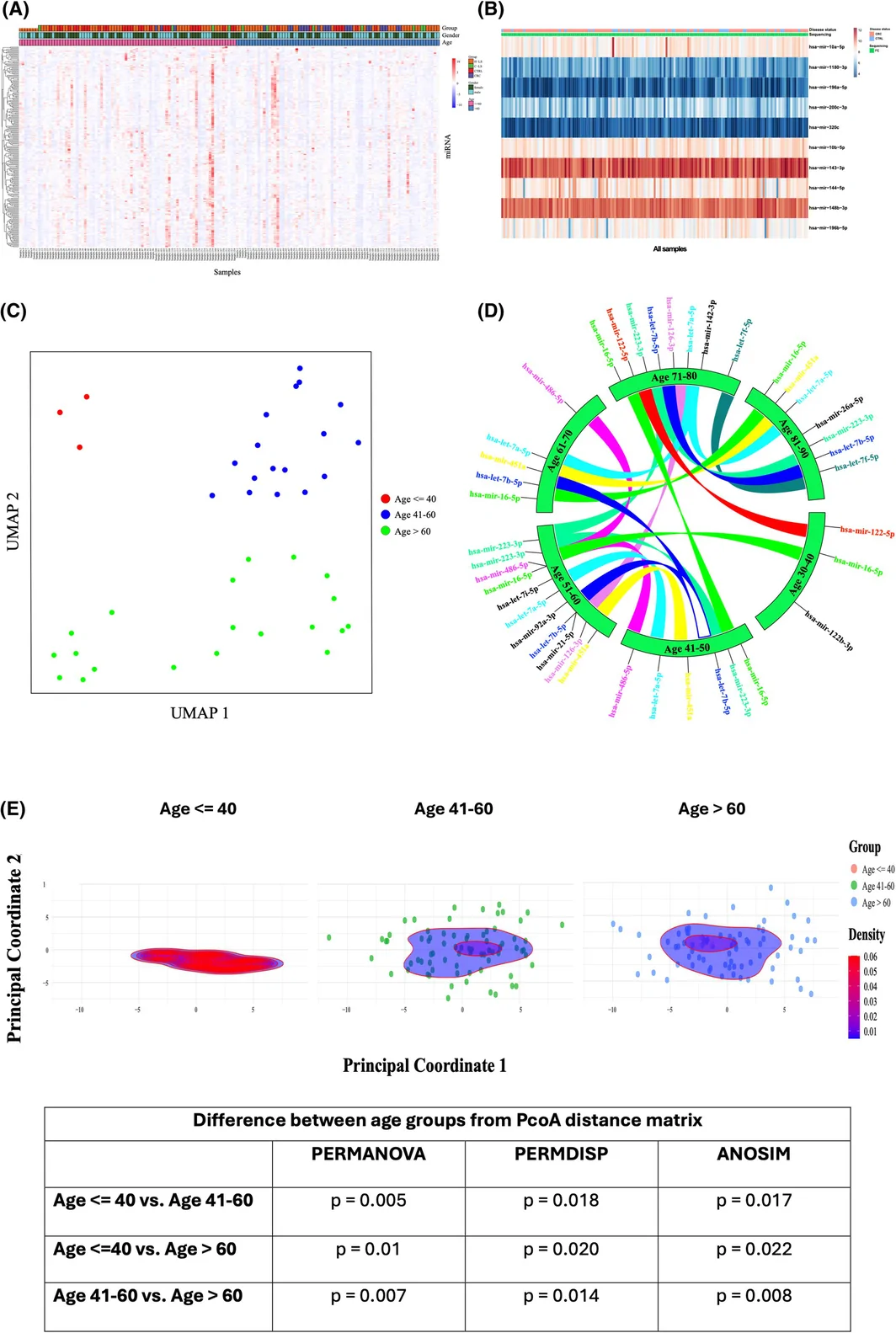
Global circulating miRNA structure by clinical group and age. (A) Hierarchical heatmap of miRNA expression across all samples, annotated by clinical group, sex and age. (B) Z‐score heatmap of the 10 miRNAs with the smallest nominal *P* values in the pooled cancer‐versus‐healthy contrast; this is a ranking display, not a claim that all 10 met the false discovery rate threshold. (C) UMAP projection colored by age group (≤ 40, 41–60 and > 60 years); each point is one participant. (D) Chord diagram linking age groups to dominant miRNA contributors. (E) Kernel‐density PCoA projections and pairwise tests of age‐group separation. PERMANOVA tests centroid separation, PERMDISP tests dispersion and ANOSIM tests rank dissimilarity; exact two‐sided *P* values are printed in the panel. ANOSIM, analysis of similarities; C‐LS, cancer‐associated Lynch syndrome; CRC, sporadic colorectal cancer; CTRL, healthy control; H‐LS, nonmalignant Lynch syndrome; miRNA, microRNA; PCoA, principal coordinates analysis; PERMANOVA, permutational multivariate analysis of variance; PERMDISP, permutational analysis of multivariate dispersions; UMAP, uniform manifold approximation and projection.

We further plotted a UMAP to stratifying these groups by age which revealed another striking trend (Fig. 2C): patients below 40 years clearly clustered together, distinct from those aged 41–60 and over 60, indicating that miRNA‐based regulatory programs undergo age‐dependent reconfiguration, the input date are shown in Table S33. To further probe these dynamics, we constructed a circos plot of dominant miRNAs across age brackets (Fig. 2D). We observed strong age‐associated transitions in miRNA wiring, with multiple age groups.

Finally, using a PCoA plot and multivariate dispersion tests (Fig. 2E), we observed statistically significant differences in miRNA composition between age groups (PERMANOVA *P* = 0.001; ANOSIM *P* = 0.001). The observed separation implies that miRNA profiles are not only reflective of disease state but are dynamically sculpted by host age, indicating that age‐specific miRNA signatures may enhance biomarker precision for LS screening and cancer risk stratification [79, 80].

### Circulating miRNAome signatures reveal age‐specific and disease‐specific molecular reprogramming

To elucidate how circulating miRNAs capture the transition from genetic susceptibility to malignancy, we systematically analyzed differential miRNA expression across diagnostic groups: CTRL, H‐LS, C‐LS, and CRC while stratifying by age. This integrated approach was designed to reveal both disease‐specific and age‐specific regulatory signatures defining the molecular continuum of carcinogenesis (Figs S7–S16).

In comparing CRC patients with CTRL (Fig. 3A), we identified a robust shift in miRNA expression, typified by the significant upregulation of miR‐183‐5p, miR‐486‐5p, and miR‐let‐7b‐5p. These members of the miR‐183/96/182 cluster are known oncogenic regulators of epithelial–mesenchymal transition and Wnt/β‐catenin signaling [81, 82, 83, 84]. Conversely, miRNAs with established tumor‐suppressive roles, such as miR‐126‐3p, miR‐27a‐3p, and miR‐10b‐3p, were markedly downregulated, consistent with epigenetic silencing mechanisms frequently observed in CRC [85, 86, 87].

**Fig. 3:**
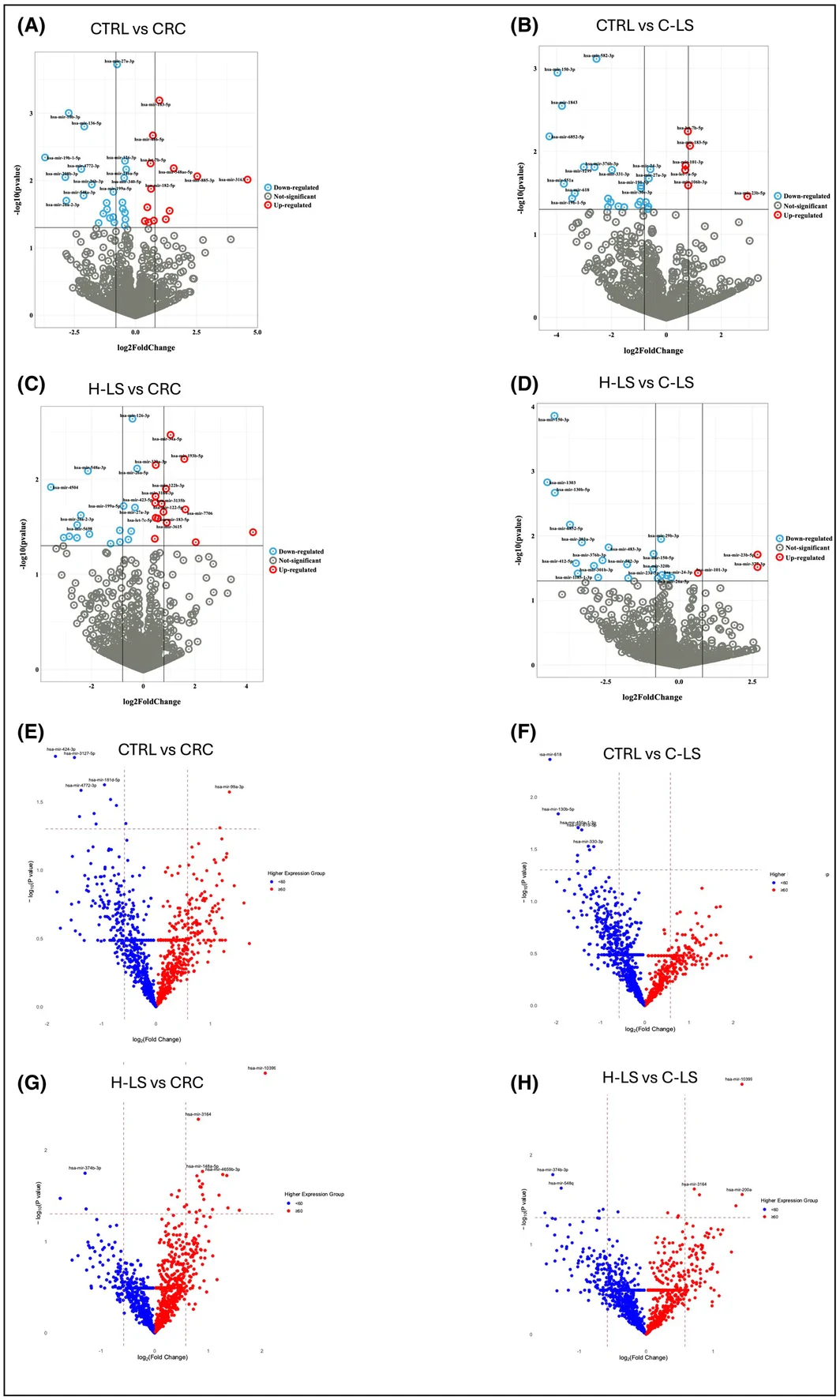
Disease‐ and age‐stratified differential circulating miRNA expression. (A–D) Volcano plots for CTRL versus CRC (A), CTRL versus C‐LS (B), H‐LS versus CRC (C), and H‐LS versus C‐LS (D). (E–H) Age‐stratified volcano plots for the corresponding disease contrasts. Each point is one miRNA. Red and blue points indicate significant upregulation and downregulation, respectively; gray points are not significant. Significance was defined as an FDR‐adjusted *P* value < 0.05 and absolute log2 fold change ≥ 1; dashed lines show these cut‐offs. For E–H, red denotes higher expression in participants aged ≥ 60 years and blue denotes higher expression in those aged < 60 years. C‐LS, cancer‐associated Lynch syndrome; CRC, sporadic colorectal cancer; CTRL, healthy control; FDR, false discovery rate; H‐LS, nonmalignant Lynch syndrome; miRNA, microRNA.

When comparing CTRL to C‐LS cases (Fig. 3B), we observed a partially overlapping miRNAome with the CRC group but also detected unique miRNAs, including the upregulation of miR‐101‐3p and miR‐106b‐3p. These findings suggest that in C‐LS, early malignant transformation may be governed by distinct regulatory circuits modulated by germline MMR mutations [63, 88]. In contrast, the H‐LS group (H‐LS vs CRC; Fig. 3C) exhibited a more immune‐regulatory and suppressive miRNA profile, with higher expression of miR‐126‐3p and miR‐199a‐5p and downregulation of oncogenic miR‐423‐5p, potentially reflecting preserved immune surveillance and genomic stability [89]. (Fig. S17).

The direct comparison between H‐LS and C‐LS (Fig. 3D) revealed several miRNAs that may mark the transition from a premalignant to malignant LS phenotype. Notably, miR‐23b‐5p, miR‐377‐3P, and miR‐101‐3p were upregulated, whereas miR‐150‐3p and miR‐130b‐5p were downregulated. These transitions may represent the molecular tipping point at which immune containment fails and tumorigenic signaling dominates, even within the same germline background [90]. (Figs S18 and S19).

Age‐specific miRNAome changes further refined our understanding of this molecular landscape. In CRC patients, individuals above 60 years of age exhibited high expression levels of miR‐99a‐3p (Fig. 3E), while younger patients (<60 years of age) showed preferential upregulation of miR‐424‐3p, miR‐3127‐5p, and miR‐181d‐5p, these miRNAs linked to DNA repair modulation and interferon signaling [91, 92]. A similar divergence emerged in the CTRL versus C‐LS group (Fig. 3F), where only younger individuals overexpressed microRNAs such as miR‐618, miR‐130b‐5p, and miR‐330‐3p, suggesting age modifies the penetrance or trajectory of miRNA‐driven pathways [93]. In H‐LS versus CRC (Fig. 3G), miR‐10399 and miR‐3164 were predominantly upregulated in older individuals. Likewise, in the H‐LS versus C‐LS comparison stratified by age (Fig. 3H), we observed miR‐10399, miR‐200a, and miR‐3164 were highly overexpressed in older patients (Figs S20–S22).

These analyses provide compelling evidence that the circulating miRNAome is not static but evolves with age and disease status, acting as a dynamic molecular recorder of cellular homeostasis, genomic stress, and immune regulation [94, 95]. Our results emphasize the potential roles of circulating miRNAs both as discriminatory patterns and mechanistic surrogates of disease progression in LS and CRC. Full disease‐contrast results are provided in Tables S21–S24, and target gene and miRNA‐target interaction outputs are provided in Tables S25–S32.

### MMR genes show distinct circulating miRNAome heterogeneity in LS patients

Because MMR gene‐resolved groups were small, particularly after stratification by cancer status, this analysis was treated as exploratory and intended to describe candidate gene‐associated miRNA patterns rather than definitive MMR gene‐specific signatures. The expanded gene‐resolved analysis revealed that the circulating miRNAome varies substantially across *MLH1*, *MSH2*, and MSH6 associated LS genes. There were several differential expression contrasts between healthy LS variant heterozygotes and cancer cases. The results indicate that the degree of circulating miRNA repression scales with the gene‐specific penetrance observed clinically, being most pronounced in *MLH1* and least in *MSH6* LS variant heterozygotes. (Fig. 4A–C). The *MLH1*‐related cancers exhibited a broad, asymmetric repression of circulating miRNAs, indicating a global loss of regulatory control consistent with a DNA repair‐deficient phenotype compared to *MLH1* healthy LS variant heterozygotes (Fig. 4A). In contrast, *MSH2*‐associated cancers showed a coherent, high‐magnitude upregulation of multiple miRNAs relative to healthy *MSH2* LS variant heterozygotes, suggesting widespread transcriptional silencing accompanying malignant transformation (Fig. 4B). In *MSH6*‐deficient cancers, distribution shows a modest right‐skew, indicating that a limited number of circulating miRNAs are upregulated in MSH6‐related cancers relative to healthy *MSH6* LS variant heterozygotes, while the majority remain unchanged (Fig. 4C).

**Fig. 4:**
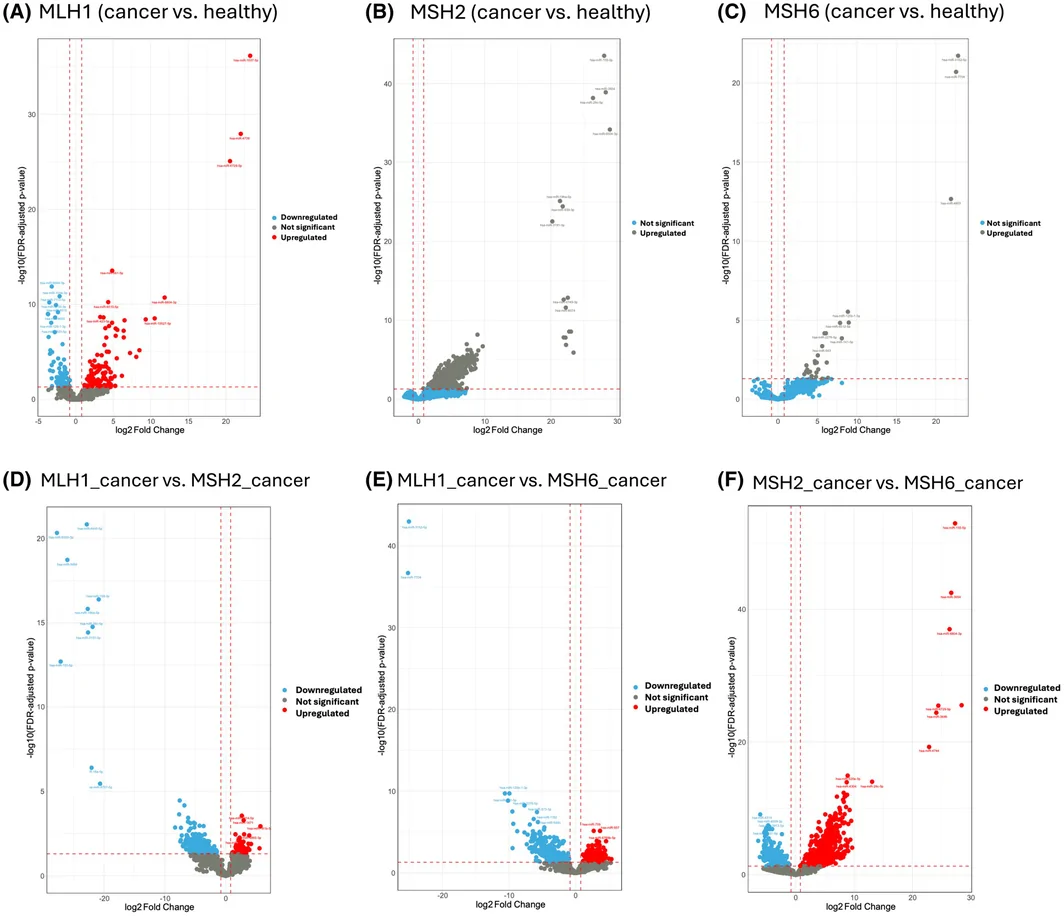
Mismatch‐repair‐gene‐specific circulating miRNA signatures. Volcano plots compare cancer with nonmalignant carriers for *MLH1* (A), *MSH2* (B) and *MSH6* (C), and compare cancers associated with *MLH1* versus *MSH2* (D), *MLH1* versus *MSH6* (E), and *MSH2* versus *MSH6* (F). Each point is one miRNA; red, blue and gray denote significant upregulation, significant downregulation and no significant change, respectively. Positive log2 fold change indicates higher expression in the first‐named group in each panel title. Dashed vertical lines mark absolute log2 fold change = 1 and the horizontal line marks FDR‐adjusted *P* = 0.05. FDR, false discovery rate; LS, Lynch syndrome; miRNA, microRNA; MLH1, MutL homolog 1; MSH2, MutS homolog 2; MSH6, MutS homolog 6.

Direct inter‐gene comparisons (Fig. 4D–F) further highlighted distinct molecular trajectories among MMR genes. The MLH1 versus MSH2 contrast (Fig. 4D) revealed strong left‐skewed differentials, signifying that MLH1 cancers retain higher circulating miRNA expression than MSH2 counterparts, consistent with greater miRNA silencing within MSH2‐defective tumors. The MLH1 versus MSH6 comparison (Fig. 4E) showed widespread enrichment of repressed transcripts in MLH1, indicating more extensive systemic deregulation compared with the relatively quiescent MSH6 profile. Conversely, the comparison between MSH2 versus MSH6 cancers (Fig. 4F) was markedly right‐skewed, dominated by upregulated signals in MSH2 cancers, suggesting stronger miRNA activation linked to immune‐inflammatory circuitry. These cross‐gene contrasts emphasize a continuum of miRNA deregulation across MMR defects from the highly repressive MLH1 state to the immunoreactive MSH2 signature and the attenuated MSH6 pattern. Although exploratory and constrained by small subgroup numbers, these results provide a descriptive overview of possible circulating miRNAome in LS. They imply that each MMR mutation may confer a distinct systemic regulatory phenotype, reflecting underlying differences in tumor biology, immune signaling and tissue tropism [96, 97].

### miRNA expression dynamics distinguish LS from CRC

To further dissect the discriminatory power of miRNA expression, we performed multivariate distance‐based analysis to evaluate group‐level divergence. PCoA plots (Fig. 5) clearly demonstrated that samples clustered according to disease subtype. C‐LS, CRC, H‐LS and CTRL each formed partially overlapping but distinct density contours, suggesting unique miRNA expression landscapes. CRC samples clustered tightly with a high‐density centroid, indicating low within‐group dispersion. In contrast, H‐LS and C‐LS samples exhibited broader, more diffuse spread with lower kernel‐density intensity, suggesting higher transcriptional variability. CTRL samples formed a compact but somewhat elongated cluster, indicating moderate dispersion. In multivariate statistical testing of group‐wise distances confirmed significant differences across all pairwise comparisons. PERMANOVA indicated differences in group centroids (H‐LS vs C‐LS *P* = 0.004, H‐LS vs CRC *P* = 0.008, CRC vs CTRL *P* = 0.007). PERMDISP supported significant differences in variance (C‐LS vs CTRL *P* = 0.010, CRC vs CTRL *P* = 0.012). ANOSIM also confirmed group‐level dissimilarity (H‐LS vs CRC *P* = 0.005, C‐LS vs CRC *P* = 0.010), with overall *P* values consistently < 0.016, validating structural and dispersion shifts in miRNA profiles. This suggests that molecular perturbations involving miRNA dysregulation emerge earlier and more robustly in C‐LS. Rather than a delayed onset, these findings imply a more aggressive or early‐primed transcriptional reprogramming in C‐LS, likely reflecting the inherent genomic instability associated with mismatch repair deficiency [69, 98]. These results reinforce the distinct biological trajectories between hereditary and CRC and support the utility of miRNA‐based biomarkers for early risk stratification in LS.

**Fig. 5:**
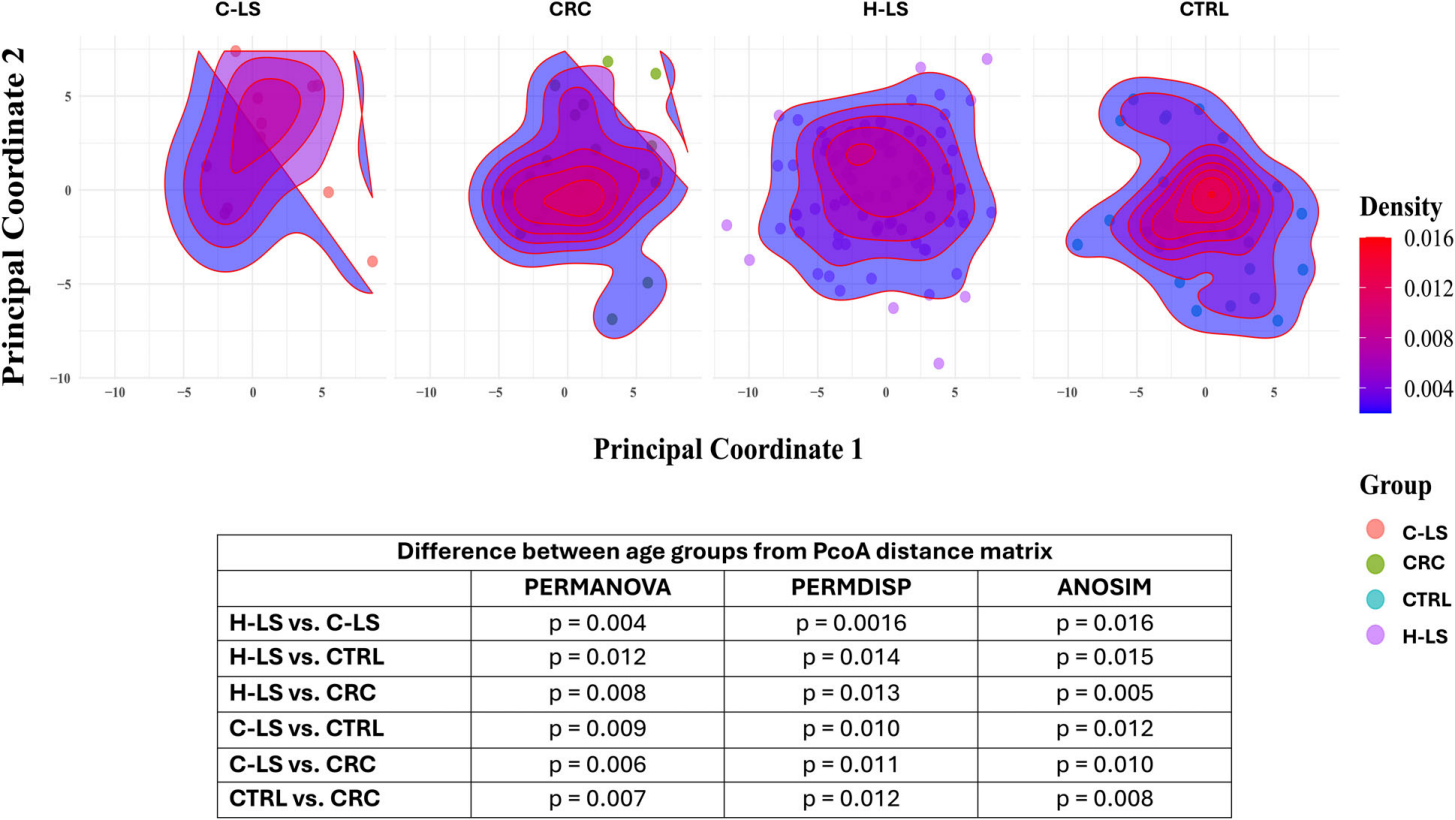
Circulating miRNA variation and clinical stratification across Lynch syndrome and sporadic colorectal cancer. Kernel‐density‐enhanced PCoA projections show C‐LS, CRC, CTRL and H‐LS samples; each point is one participant and density contours summarize within‐group concentration. Pairwise PERMANOVA tests centroid separation, PERMDISP tests dispersion and ANOSIM tests rank dissimilarity. Exact *P* values are printed in the accompanying table and no asterisk coding is used. ANOSIM, analysis of similarities; C‐LS, cancer‐associated Lynch syndrome; CRC, sporadic colorectal cancer; CTRL, healthy control; H‐LS, nonmalignant Lynch syndrome; miRNA, microRNA; PCoA, principal coordinates analysis; PERMANOVA, permutational multivariate analysis of variance; PERMDISP, permutational analysis of multivariate dispersions.

### Age‐associated miRNA expression dynamics in cancer and healthy controls

To define the relationship between chronological age and miRNA expression specifically within the malignant context, we next analyzed expression trajectories of 12 significantly overexpressed miRNAs in cancer patients (Fig. 6A). A consistent pattern of age‐associated dysregulation emerged, marked by nonlinear shifts in expression across the aging spectrum. Among these, hsa‐miR‐132‐3p and hsa‐miR‐598‐3p demonstrated robust inverse correlations with age (*r* = −0.221 and − 0.209, respectively; *P* < 0.03), suggesting potential downregulation of tumor‐modulatory pathways in aged patients. Notably, hsa‐miR‐181a‐3p, a known regulator of immune responsiveness, also showed significant age‐linked decline (*r* = −0.203, *P* = 0.0265), highlighting potential immunomodulatory rewiring in aging tumors. Conversely, miRNAs such as hsa‐miR‐22‐3p and hsa‐miR‐532–5p displayed positive correlations with age, though of modest magnitude (*r* = 0.184 and 0.198), suggesting context‐specific age‐associated upregulation that may reflect compensatory transcriptional buffering or oncogenic reprogramming. Interestingly, while several miRNAs such as let‐7c‐5p and miR‐625‐5p did not reach statistical significance, their consistent negative trajectories raise the possibility of subthreshold trends that warrant further validation. These results reveal a transcriptional axis of age‐responsive miRNAs in cancers.

**Fig. 6:**
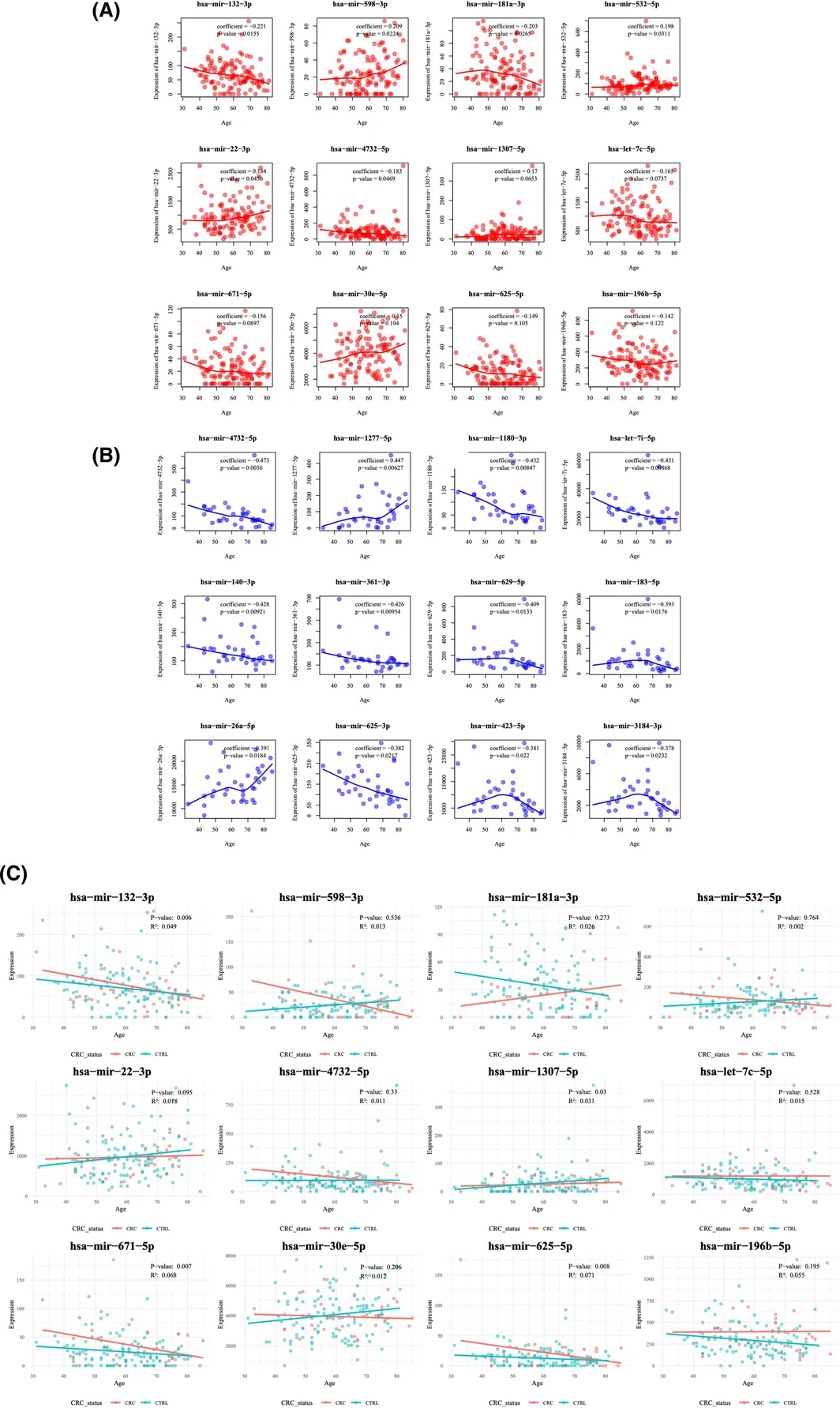
Age‐associated circulating miRNA expression in cancer and healthy controls. (A) Expression‐versus‐age relationships in participants with cancer. (B) Corresponding relationships in healthy controls. (C) Direct comparison of age‐related trajectories in CRC and CTRL. Points represent participants, lines are ordinary least‐squares fits and shaded bands are 95% confidence intervals. In A and B, two‐sided *P* values test the age coefficient. In panel C, models included age, disease status and their interaction; displayed two‐sided *P* values test the age × disease‐status interaction and R‐squared is the coefficient of determination for the full model. CRC, sporadic colorectal cancer; CTRL, healthy control; hsa, Homo sapiens; miRNA/miR, microRNA.

To distinguish miRNA aging trajectories intrinsic to physiological aging from those shaped by malignancy, we next evaluated expression‐age relationships in healthy control individuals. A focused panel of 12 significantly overexpressed miRNAs was assessed for correlation with age across the control cohort (Fig. 6B), revealing a striking and coherent pattern of age‐associated transcriptional drift. The majority of miRNAs exhibited significant negative correlations with age, including hsa‐miR‐4732‐5p, miR‐1180‐3p, miR‐140‐3p, miR‐361‐3p, and miR‐3184‐3p (all *P* < 0.01), suggesting a progressive silencing of these transcripts across the aging continuum. Notably, miR‐4732‐5p and miR‐1180‐3p demonstrated the strongest associations (*r* = −0.473 and −0.432, respectively), pointing to robust age sensitivity in the absence of disease. Several of these miRNAs including let‐7i‐5p, miR‐183‐5p, and miR‐629‐5p have previously been implicated in epithelial differentiation, immune surveillance, or stress signaling, raising the possibility that their age‐linked suppression reflects broader remodeling of homeostatic regulatory circuits with advancing age [99, 100]. In contrast, miR‐1277‐5p was the only transcript displaying a significant positive correlation with age (*r* = 0.447, *P* = 0.006), suggesting selective miRNA activation as a compensatory or adaptive mechanism in aging tissues [22, 101].

To determine whether age‐associated miRNA expression patterns diverge between malignant and nonmalignant states, we directly compared the age‐expression trajectories of 12 selected miRNAs significantly correlated in Fig. 6B for cancer patients versus healthy controls. This comparative framework enabled us to dissect the extent to which tumor biology modulates age‐linked transcriptional shifts (Fig. 6C). Strikingly, several miRNAs that displayed age‐associated decline in healthy controls exhibited blunted or inverted trajectories in cancer, indicating a decoupling of canonical aging signals in the tumor context. For instance, hsa‐miR‐671‐5p and hsa‐miR‐625‐5p exhibited stronger negative age correlations in CRC patients compared to controls, with CRC‐specific slopes indicating a steeper decline in expression with advancing age (*P* = 0.007 and *P* = 0.008, respectively). Conversely, hsa‐miR‐1307‐5p, which showed minimal correlation with age in controls, exhibited a weak but statistically significant increase with age in CRC (*P* = 0.03), hinting at cancer‐specific age‐related activation. Similarly, miR‐132‐3p, a regulator of cellular senescence and inflammation, retained a consistent negative trend in both cohorts, but the slope was accentuated in CRC samples (*P* = 0.006), suggesting enhanced age sensitivity in tumors [102, 103]. Other miRNAs, such as miR‐598‐3p and let‐7c‐5p, exhibited no meaningful age association in either group, indicating selectivity in the aging response landscape. Our data highlight a subset of miRNAs whose expression patterns are both age‐sensitive and disease‐specific, providing a rational basis for their further development as biomarkers for early cancer detection and LS molecular surveillance [104, 105].

### Functional enrichment of CRC‐associated miRNAs

To elucidate the biological relevance of circulating miRNAs differentially expressed in CRC, we performed multi‐layered enrichment analyses across six curated databases. We analyzed an overview of pathway enrichment with the largest number and magnitude of significant terms derived from the Gene Ontology (GO) database (blue), followed by the KEGG database (orange) and Reactome database (green). BioCarta, PID, and ImmuneSigDB revealed broader and consistent enrichments in CRC compared to CTRL and C‐LS compared to H‐LS patients suggesting a distinct reprogramming of miRNA‐regulated pathways during malignant progression (Fig. 7A,B). This landscape illustrates that CRC‐associated miRNAs converge on conserved biological systems captured across databases [106].

**Fig. 7:**
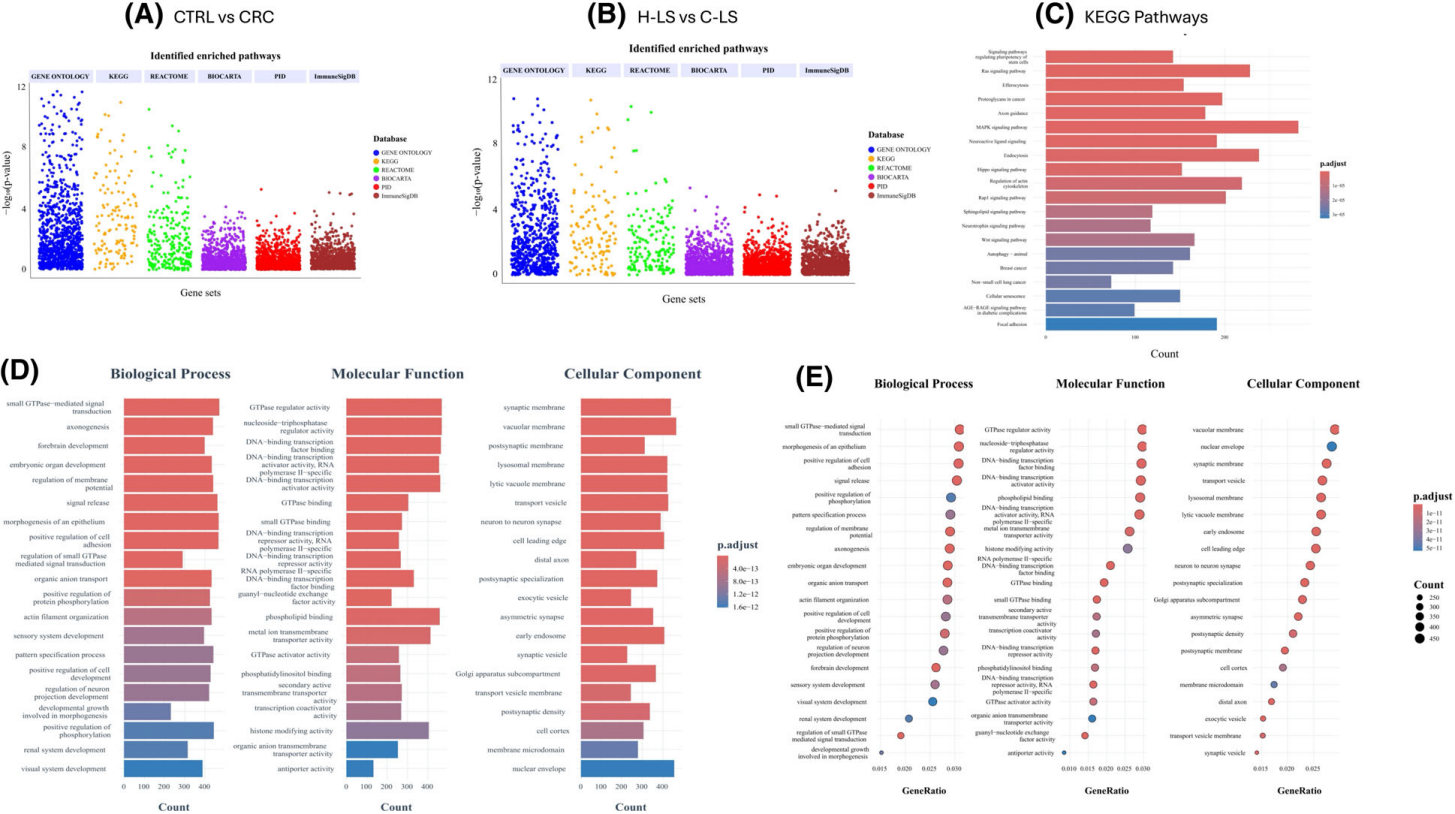
Functional enrichment of predicted and validated targets of cancer‐associated circulating miRNAs. (A, B) Enriched terms across GO, KEGG, Reactome, BioCarta, PID and ImmuneSigDB for CRC versus CTRL and C‐LS versus H‐LS target sets; position shows −log10(P), and color identifies the database. (C) The 20 highest‐ranked KEGG pathways; bar length is gene count and color is the Benjamini‐Hochberg‐adjusted *P* value. (D) GO biological process, molecular function and cellular‐component terms. (E) GO bubble plots; the *x*‐axis is GeneRatio, bubble size is gene count and color is adjusted *P* value. C‐LS, cancer‐associated Lynch syndrome; CRC, sporadic colorectal cancer; CTRL, healthy control; GO, Gene Ontology; H‐LS, nonmalignant Lynch syndrome; ImmuneSigDB, Immunologic Signature Gene Set Database; KEGG, Kyoto Encyclopedia of Genes and Genomes; miRNA, microRNA; PID, Pathway Interaction Database.

Among the top‐ranked pathways were canonical cancer regulators including MAPK signaling, Wnt signaling, Ras signaling, proteoglycans in cancer and focal adhesion (Fig. 7C). Developmental and neural pathways such as axon guidance, neuroactive ligand‐receptor interaction and Hippo signaling were also significantly enriched, along with senescence‐related and metabolism‐related pathways including autophagy, AGE‐RAGE signaling, and cellular senescence. Using GO enrichment splitted by domains: (i) Biological Process (BP), (ii) Molecular Function (MF) and (iii) Cellular Component (CC), the BP terms emphasized on axonogenesis, forebrain development, GTPase‐mediated signaling, and actin filament organization. This indicated developmental reactivation and cytoskeletal remodeling (Fig. 7D). MF terms highlighted DNA‐binding transcription factor activity, GTPase regulation and chromatin remodeling, suggesting strong influence on gene regulatory networks. CC terms revealed enrichment in synaptic membranes, endosomal vesicles, nuclear envelopes and lysosomal compartments, indicating miRNA‐mediated control over membrane trafficking and cellular compartmentalization. Upon visualizing the same GO domains using bubble plots, larger bubbles with higher gene ratios and lower adjusted *P* values clustered around developmental (e.g., morphogenesis, axonogenesis) and transcriptional processes (e.g., RNA polymerase II‐specific binding, histone modification), with a prominent presence of vesicular and synaptic structures in the CC domain (Fig. 7E). The consistent targeting of neuronal, epithelial and intracellular transport mechanisms reinforces the hypothesis that CRC miRNAs co‐opt embryonic signaling programs and spatial organization systems to support tumor progression. The enrichment patterns across Fig. 7 demonstrate that circulating miRNAs dysregulated in CRC are functionally linked to core developmental, transcriptional and subcellular organization programs, offering mechanistic insight into their role in tumor evolution, cellular plasticity, and intercellular communication [63, 107]. Target gene lists are provided in Tables S25 and S26.

### miRNA pathway enrichment and transcriptomic structure differ across LS, CRC, and controls

To elucidate the functional architecture and intergroup variation in circulating miRNA signatures, we performed an integrated pathway enrichment and dimensionality reduction analysis across all four clinical categories: H‐LS, C‐LS, CRC, and CTRL. Fig. 8A presents the enrichment landscape based on miRNAs differentially expressed for upregulated genes between H‐LS and C‐LS. The comprehensive dot plot spans six major knowledge bases: (i) GO, (ii) KEGG, (iii) Reactome, (iv) BioCarta, (v) PID, and (vi) ImmuneSigDB highlighting a dense distribution of highly enriched pathways. The GO terms (blue) were overwhelmingly dominant, with numerous biological processes related to signal transduction, immune regulation, and development reaching extremely high statistical significance (−log_10_ *P* > 10). KEGG and Reactome pathways (such as MAPK, Wnt, and Ras signaling) were also strongly enriched.

**Fig. 8:**
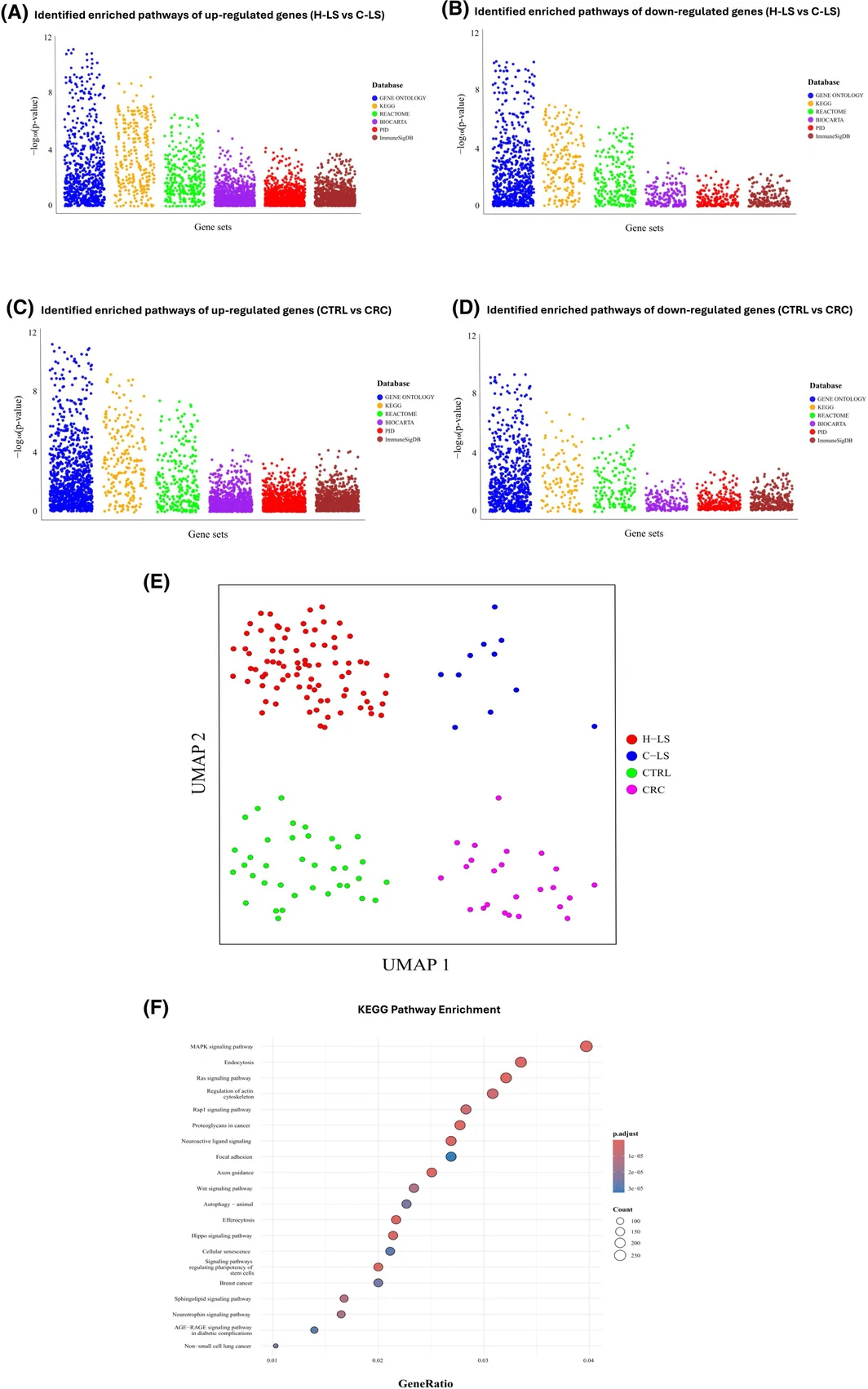
Enrichment and low‐dimensional structure of circulating miRNAs across disease states. (A, B) Enrichment of targets of miRNAs upregulated (A) or downregulated (B) in C‐LS relative to H‐LS across GO, KEGG, Reactome, BioCarta, PID and ImmuneSigDB. (C, D) Corresponding enrichment for miRNAs upregulated (C) or downregulated (D) in CRC relative to CTRL. (E) UMAP projection of sample‐level miRNA expression across H‐LS, C‐LS, CRC and CTRL; each point is one participant. (F) The 20 highest‐ranked KEGG pathways across comparisons; the x‐axis is GeneRatio, bubble size is gene count and color is the Benjamini‐Hochberg‐adjusted *P* value. C‐LS, cancer‐associated Lynch syndrome; CRC, sporadic colorectal cancer; CTRL, healthy control; GO, Gene Ontology; H‐LS, nonmalignant Lynch syndrome; ImmuneSigDB, Immunologic Signature Gene Set Database; KEGG, Kyoto Encyclopedia of Genes and Genomes; miRNA, microRNA; PID, Pathway Interaction Database; UMAP, uniform manifold approximation and projection.

Fig. 8B extends this analysis to compare for downregulated genes between H‐LS and C‐LS, two clinically similar but genetically distinct LS populations. Again, GO emerged as the most heavily enriched database, followed by KEGG and Reactome. However, the overall distribution of *P* values was more moderate than in the upregulated genes, suggesting subtler but biologically meaningful distinctions. We observed a lower immune‐related pathways from ImmuneSigDB in upregulated genes analysis compared to upregulated genes analysis (8A versus 8B), indicating that while both groups share core LS features, there are distinct functional alterations.

In the comparison between CTRL and CRC patients, pathway enrichment analysis of upregulated genes revealed dominant involvement of core biological processes, particularly within GO and KEGG databases (Fig. 8C). These included pathways related to cell proliferation, adhesion, and metabolic activation, reflecting hallmark features of tumorigenesis.

Pathway enrichment analysis of downregulated genes in CTRL versus CRC revealed strong enrichment in GO and KEGG databases as well, with reduced involvement of immune‐related pathways such as PID, BIOCARTA, and ImmuneSigDB (Fig. 8D). The suppressed genes were predominantly linked to normal cellular homeostasis, differentiation, and metabolic regulation, suggesting that CRC progression is accompanied by transcriptional silencing of essential physiological functions. The lack of immune signaling enrichment further indicates that immune evasion in CRC may arise more from active suppression mechanisms rather than broad downregulation of immune gene expression.

To visualize group‐level transcriptomic structure, Fig. 8E shows the UMAP embedding of circulating miRNA profiles according to disease groups, the input date is shown in Table S34. Strikingly, each of the four groups, H‐LS (red), C‐LS (blue), CRC (purple), and CTRL (green) formed discrete and nonoverlapping clusters. This unsupervised separation shows the robustness of circulating miRNA expression as a group‐level discriminator. H‐LS samples were tightly clustered, suggesting homogeneity likely driven by germline MMR mutations. C‐LS samples were more dispersed, consistent with clinical heterogeneity. CRC and CTRL groups formed distinct clusters, affirming the transcriptomic divergence of cancer vs. noncancer states.

To focus on biologically actionable mechanisms, Fig. 8F provides a KEGG pathway bubble plot highlighting the 20 most enriched terms. The analysis revealed substantive enrichment of canonical cancer‐related pathways, including MAPK signaling, Ras signaling, Wnt signaling, and endocytosis, each with substantial gene counts and highly significant adjusted *P* values. Notably, pathways related to autophagy, focal adhesion, cellular senescence, and proteoglycans in cancer were also prominent, reinforcing the involvement of miRNA regulation in both oncogenesis and immune modulation. The presence of AGE‐RAGE and neuroactive ligand signaling pathways suggests broader systemic involvement, potentially linking cancer progression to metabolic and inflammatory dysregulation [108, 109]. Fig. 8 reveals a coordinated rewiring of regulatory pathways captured via circulating miRNAs across disease states. While CRC and H‐LS both show deep alterations in key oncogenic and immunologic programs, the divergence between H‐LS and C‐LS suggests that miRNA signatures carry biologically meaningful information beyond clinical classification. These results demonstrate the power of integrative miRNA‐based systems biology in distinguishing disease etiology, progression, and risk and pave the way for miRNA‐based diagnostics and patient stratification tools.

### Regulatory miRNA‐gene network landscape reveals distinct oncogenic and hereditary signatures across CRC and LS

To delineate how miRNA‐mRNA regulatory interactions shape disease phenotypes and age trajectories, we constructed comprehensive interaction networks integrating differential miRNA expression with experimentally validated and predicted target genes. This systems‐level approach revealed distinct regulatory architectures distinguishing CTRL, H‐LS, C‐LS, and CRC, while also capturing the temporal dynamics of aging. We began by comparing healthy controls and CRC cases (Fig. 9A), thus uncovering a densely interconnected miRNA‐mRNA network indicative of widespread post‐transcriptional deregulation in cancer. We observed highly connected miRNAs suggesting key regulatory roles. The dense network implies significant dysregulation of gene expression by specific miRNAs in CRC pathogenesis [110, 111].

**Fig. 9:**
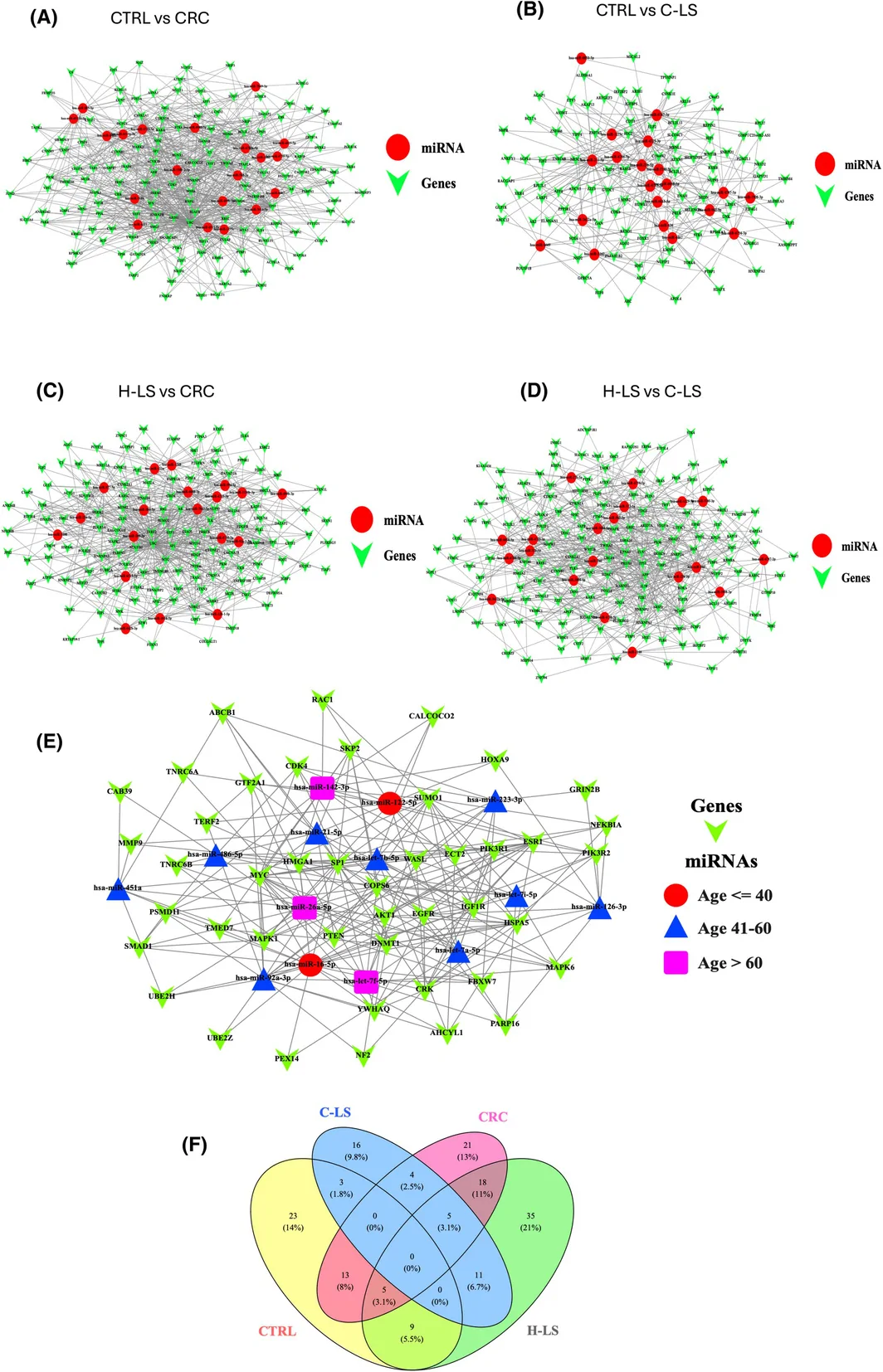
Disease‐ and age‐stratified miRNA‐mRNA interaction networks. Networks compare CTRL with CRC (A), CTRL with C‐LS (B), H‐LS with CRC (C) and H‐LS with C‐LS (D). Red circles are miRNAs, green arrowheads are target genes and gray edges are high‐confidence regulatory interactions retained from TargetScan or miRTarBase. (E) Age‐stratified interaction network; red circles, blue triangles and magenta squares denote miRNAs associated with ages ≤ 40, 41–60, and > 60 years, respectively; green arrowheads are target genes. (F) Venn diagram of unique and shared differentially expressed miRNAs across CTRL, H‐LS, C‐LS and CRC. C‐LS, cancer‐associated Lynch syndrome; CRC, sporadic colorectal cancer; CTRL, healthy control; H‐LS, nonmalignant Lynch syndrome; miRNA, microRNA; mRNA, messenger RNA.

Next, contrasting CTRL with C‐LS (Fig. 9B) revealed a more modular network. A moderately interconnected regulatory landscape was observed, comprising several key miRNAs targeting a distinct set of genes. Unlike the dense and globally dysregulated network seen in CRC, the C‐LS network demonstrated a more focused architecture, with fewer miRNA‐gene connections and reduced node density. This pattern suggests a targeted dysregulation of specific genes, potentially reflecting the underlying MMR deficiency characteristic of LS [112, 113].

In the comparison between H‐LS and CRC (Fig. 9C), a densely interconnected regulatory structure was observed, with numerous miRNAs targeting a broad array of genes. This network resembled the global dysregulation, indicating that H‐LS individuals, despite lacking overt malignancy, harbor extensive miRNA‐mediated transcriptomic alterations. This suggests their early involvement in modulating oncogenic pathways. These findings imply that healthy LS variant heterozygotes may exhibit preclinical miRNA perturbations that predispose to cancer development, offering a potential window for biomarker‐based surveillance. Also, they support the notion that certain miRNAs may function as molecular ‘gatekeepers’, delaying or mitigating malignant conversion in genetically predisposed individuals [114]. From the H‐LS vs C‐LS network (Fig. 9D), a moderately dense regulatory topology was observed, indicating substantial miRNA‐mediated transcriptomic reprogramming during malignant progression. While the overall connectivity remained less extensive than in CRC, this network reveals distinct regulatory shifts specific to LS‐driven tumorigenesis and highlights potential miRNA biomarkers indicative of early malignant transformation.

To integrate temporal trends with disease phenotypes, we constructed an age‐stratified regulatory network (Fig. 9E). In the age‐stratified miRNA‐gene interaction network, differential regulatory patterns emerged across three age groups, revealing distinct age‐associated miRNA signatures potentially modulating cancer biology. Notably, individuals aged 41–60 years of age (blue triangles) exhibited the highest miRNA‐gene connectivity, implicating this age window as a critical period for miRNA‐mediated transcriptomic shifts. In contrast, miRNAs from younger (≤ 40 years of age, red circles) and older (> 60 years of age, pink squares) individuals displayed a fewer but strategically positioned interactions, targeting key oncogenic regulators such as MYC, and EGFR. The clustering of central signaling genes around age‐specific miRNAs suggests that age not only influences miRNA expression but also shapes the architecture of gene regulatory networks, underscoring its potential role in modulating disease trajectory and therapeutic responsiveness [115, 116].

Finally, to capture the number of group‐specific and shared miRNA signatures, we generated a four‐ways Venn diagram illustrating the distribution of differentially expressed miRNAs across the four groups (Fig. 9F). Notably, the majority of miRNAs were uniquely associated with specific groups; 35 miRNAs (21%) in H‐LS, 23 (14%) in CTRL, 21 (13%) in CRC, and 16 (9.8%) in C‐LS, underscoring the molecular heterogeneity between hereditary and sporadic cancer contexts. Overlaps were rare, with only five miRNAs (3.1%) shared between H‐LS, C‐LS and CRC, and no miRNAs common to all four groups.

### External orthogonal validation of the prespecified miRNA panel in an independent primary‐tissue cohort

To test whether the principal circulating miRNA signal identified in the discovery analysis remained detectable beyond the original dataset, we performed an orthogonal external validation on the independent primary colorectal tissue cohort GSE30454 (Table S35), which included normal (*n* = 20), LS (*n* = 22), sporadic MSI (*n* = 13) and MSS (*n* = 19) samples. Cohort metadata, the six‐miRNA sample‐level expression matrix, group summaries and source‐study support for this analysis are provided in Table S35. Given the rarity of LS and the scarcity of publicly available miRNA datasets with LS‐specific annotation, this cohort was selected because it is the only currently available external dataset that enables simultaneous assessment of normal tissue, LS, sporadic MSI and MSS disease states within a single validation framework. We therefore prespecified a focused 6‐miRNA panel‐miR‐486‐5p, miR‐183, miR‐130b, miR‐598, miR‐99a, and miR‐106b‐for external testing, prioritizing markers that were biologically central to the manuscript, technically represented in the validation cohort, and supported by prior microarray and/or qPCR evidence in the source study.

Across the external cohort, this panel preserved clear multiclass structure. We showed coordinated class‐dependent shifts in expression across the six miRNAs (Fig. 10A), and the PCA projection demonstrated nonrandom separation of normal, LS, sporadic MSI and MSS samples, with normal tissues occupying the most distinct nonmalignant space and LS remaining partially separable from sporadic MSI despite both belonging to the mismatch repair‐deficient spectrum (Fig. 10B). At the aggregate level, the composite 6‐miRNA validation score differed significantly across the four classes (Kruskal–Wallis *P* < 0.001; Fig. 10C). Pairwise comparisons showed that the strongest separation involved the biologically central MSI‐related contrasts, specifically LS versus sporadic MSI (*P* < 0.001) and sporadic MSI versus MSS (*P* < 0.001), whereas normal versus LS (*P* = 0.064) and LS versus MSS (*P* = 0.073) were weaker (Fig. 10C). This pattern indicates that the external panel retains disease‐axis structure most relevant to the study's central question, rather than functioning as a generic tumor marker alone. Across the two prespecified orthogonal comparisons, the six miRNAs did not contribute uniformly: several markers showed stronger separation for LS versus sporadic MSI, whereas others contributed more strongly to the broader all tumors versus normal contrast (Fig. 10D). Consistent with this, the evidence‐support matrix showed that the validation panel was anchored not only in the external reanalysis itself but also in previously reported microarray and qPCR support across LS/MSI, MSI/MSS and CRC/normal comparison domains (Fig. 10E). In particular, miR‐130b and miR‐598 aligned with LS‐versus‐sporadic‐MSI support, miR‐99a and miR‐106b aligned with MSI/MSS‐related support, and miR‐183 and miR‐486‐5p showed additional support in qPCR validation layers, strengthening the biological and technical rationale for retaining this prespecified six‐miRNA subset for external testing. In simple terms, the same miRNAs that appeared important in our circulating miRNA discovery analysis also showed supportive disease‐related behavior in an independent tissue dataset, indicating that our main signal is unlikely to be an artifact of the original plasma cohort and instead reflects a reproducible LS‐CRC molecular‐state pattern.

**Fig. 10:**
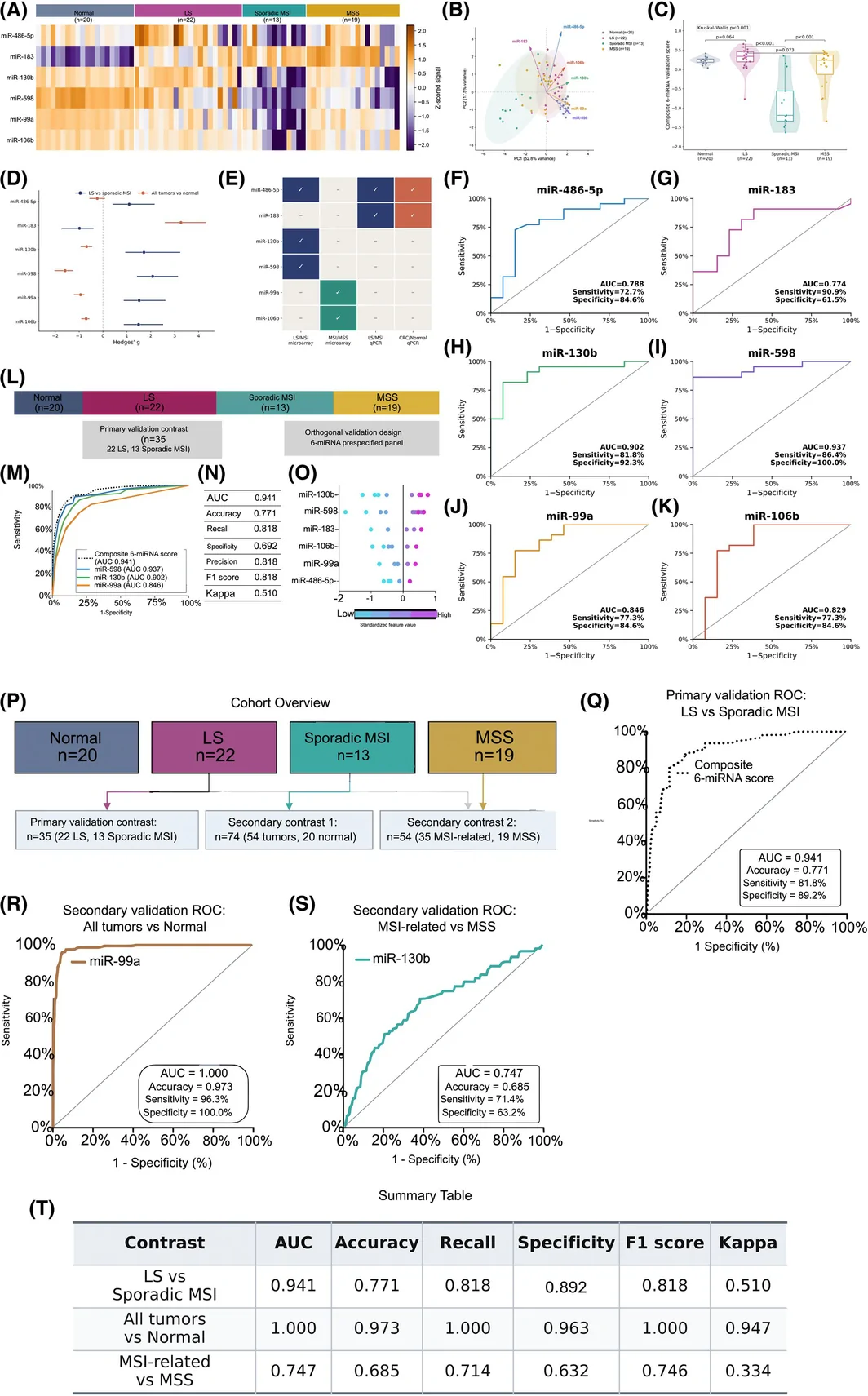
External orthogonal validation of a prespecified six‐miRNA panel in GSE30454 primary colorectal tissue. (A) Row‐wise z‐score heatmap for normal (*n* = 20), LS (*n* = 22), sporadic MSI (*n* = 13), and MSS (*n* = 19) samples. (B) PCA biplot of the same six markers; points are specimens and arrows are marker loadings. (C) Composite validation score by class. Violin width represents distribution density, points are specimens, the center line is the median, the box spans the IQR and whiskers extend to 1.5× IQR. The global Kruskal–Wallis test gave *P* < 0.001; Dunn pairwise tests gave normal versus LS *P* = 0.064, LS versus sporadic MSI *P* < 0.001, sporadic MSI versus MSS *P* < 0.001 and LS versus MSS *P* = 0.073. (D) Hedges' g standardized effect sizes for LS versus sporadic MSI and all tumors versus normal; bars are 95% confidence intervals. (E) Published‐support matrix across microarray and qPCR evidence classes. (F–K) Individual ROC curves for miR‐486‐5p, miR‐183, miR‐130b, miR‐598, miR‐99a and miR‐106b, respectively, for LS versus sporadic MSI. (L) External‐cohort overview. (M) ROC comparison: black dotted line, composite six‐miRNA score; blue line, miR‐598 (AUC 0.937); green line, miR‐130b (AUC 0.902); orange line, miR‐99a (AUC 0.846). (N) Primary model classification metrics. (O) Standardized feature contributions; sign denotes direction and magnitude denotes relative contribution. (P) Cohort and contrast overview. (Q–S) ROC curves for LS versus sporadic MSI (Q), all tumors versus normal (R), and MSI‐related tumors versus MSS (S). (T) Cross‐contrast classification metrics. AUC, area under the ROC curve; CI, confidence interval; CRC, colorectal cancer; IQR, interquartile range; LS, Lynch syndrome; miRNA/miR, microRNA; MSI, microsatellite instability; MSS, microsatellite stable; PCA, principal component analysis; qPCR, quantitative polymerase chain reaction; ROC, receiver operating characteristic.

Single‐marker ROC analyses showed that external discrimination in the primary contrast was not attributable to one feature alone. Among individual miRNAs, miR‐486‐5p yielded an AUC of 0.788 (Fig. 10F), miR‐183 an AUC of 0.774 (Fig. 10G), miR‐130b an AUC of 0.902 (Fig. 10H), miR‐598 an AUC of 0.937 (Fig. 10I), miR‐99a an AUC of 0.846 (Fig. 10J), and miR‐106b an AUC of 0.829 (Fig. 10K). These results show that several components of the prespecified panel retain measurable and reproducible discriminatory signal in the most stringent LS‐specific external tissue comparison. This supports our previously presented work by showing that the LS‐CRC molecular‐state signal is not dependent on a single miRNA or one analytical result; instead, several miRNAs from the original discovery analysis independently carried the same disease‐separating information in an external primary‐tissue cohort.

The focused validation framework is summarized in Fig. 10L. The composite ROC context panel in Fig. 10M shows that the integrated 6‐miRNA score outperformed or matched the best individual markers in the LS‐versus‐sporadic‐MSI comparison, with the composite model achieving an AUC of 0.941. The associated model summary table (Fig. 10N) showed accuracy 0.771, recall 0.818, precision 0.818, specificity 0.692, F1 score 0.818 and kappa 0.510 for the primary validation model. Feature‐contribution analysis (Fig. 10O) further indicated that the discriminatory performance of the primary model arose from coordinated multi‐marker behavior rather than a single‐feature effect, with miR‐130b, miR‐598, miR‐183, miR‐106b, miR‐99a, and miR‐486‐5p all contributing to the classification boundary in the external cohort, thus providing support for the presented results and interpretation of our data prior to this validation framework.

We then extended this analysis to broader secondary disease axes to avoid over‐reliance on a single selective comparison. The expanded cohort size overview and contrast design are shown in Fig. 10P. In the dedicated primary ROC panel for LS versus sporadic MSI (Fig. 10Q), the summary of composite 6‐miRNA score achieved an AUC of 0.941, with accuracy 0.771, sensitivity 81.8% and specificity 89.2% as displayed in the figure. In the all tumors versus normal secondary contrast (Fig. 10R), miRNA miR‐99a achieved an AUC of 1.000, accuracy 0.973, sensitivity 96.3%, and specificity 100.0%. In the MSI‐related versus MSS secondary contrast (Fig. 10S), miRNA miR‐130b performance was similarly informative, with AUC 0.747, accuracy 0.685, sensitivity 71.4%, and specificity 63.2%. Finally, the cross‐contrast summary table (Fig. 10T) showed that this validation framework defined a coherent hierarchy of performance: strongest discrimination for broad malignant‐versus‐nonmalignant biology, robust discrimination for the biologically stringent LS‐versus‐sporadic‐MSI contrast, and intermediate performance across the broader mismatch repair axis.

Our additional orthogonal analyses show that the principal disease‐axis signal identified in circulating miRNA data is externally concordant in independent primary tissue, particularly along the LS‐versus‐sporadic‐MSI and tumor‐versus‐normal dimensions, while remaining consistent with the broader MSI/MSS disease structure. We therefore interpret this validation as strong orthogonal evidence that the study's central molecular‐state signal is reproducible beyond the original circulating miRNA dataset. Accordingly, the inferences, deductions and conclusions reached in our study prior to this validation framework are further buttressed by this constructive and rigorous analysis.

## Discussion

Using a systems biology and bioinformatics approach to transcriptomics and cancer immunogenomics, we provide the first evidence suggesting that age‐associated and disease‐associated circulating miRNAome defines distinct immunogenomic regulatory states in LS and CRC. We have shown differential patterns suggesting that germline MMR mutations in LS patients shape a distinct circulating miRNAs and immunogenomic profile even in the absence of overt malignancy. These findings support the concept that miRNAs function as systemic surrogates of inherited immunogenomic architecture, offering insights into immune surveillance, tumor predisposition, and potential stratification for immune‐preventive interventions. Identification of age‐related miRNA convergence signatures exclusive to C‐LS and CRC is novel and highlights a common path to malignancy transformation that transcends genetic etiology. Our approach departs from previous approaches that focused solely on tissue expression or single‐gene alterations by integrating regulatory network architecture.

Previous studies have identified individual miRNAs associated with CRC or LS but have limited exploration of the age‐stratified regulatory depth of miRNA networks in CRC and LS [16, 17, 68, 85, 117]. Our work bridges this gap by showing age‐influenced regulatory evolution that underlies phenotypic heterogeneity in CRC and LS. This reconceptualization has implications beyond CRC, potentially informing biomarker strategies in other hereditary cancer syndromes according to age. Furthermore, our network‐based analysis shifts focus from differentially expressed miRNAs to those with high centrality and regulatory influence. This systems‐level perspective facilitates a deeper understanding of how miRNA circuits orchestrate disease progression and immune escape. It also provides a platform for identifying actionable nodes that may serve as age‐related biomarkers for cancer or therapeutic entry points. The age‐stratified miRNA profiles further support the concept of molecular aging clocks that can calibrate surveillance based on biological not chronological age. Such precision surveillance could reduce overtreatment in low‐risk individuals and enable timely intervention in high‐risk patients.

The external orthogonal validation analysis strengthens the interpretation of the discovery findings while also defining their evidentiary boundary. In GSE30454, an independent primary colorectal tissue cohort, a prespecified six‐miRNA panel preserved disease‐axis structure across normal, LS, sporadic MSI and MSS samples. The most stringent primary contrast was LS versus sporadic MSI, because both groups are mismatch repair‐deficient and therefore biologically closer than tumor‐versus‐normal comparisons. The composite panel showed strong discrimination in this setting and retained broader structure across malignant‐versus‐normal and MSI‐related‐versus‐MSS axes. These results support external concordance of the central molecular‐state signal, but they do not establish a clinically validated blood‐based assay because the validation cohort used primary tissue rather than plasma and was not prospectively collected.

Integrative network analysis uncovers a profound systems‐level distinction in how miRNA‐mRNA interactions are organized across clinical subtypes, with immunologic buffering dominating H‐LS and oncogenic hub activation defining CRC. In H‐LS, several microRNAs target key oncogenes and maintain regulatory equilibrium, forming a resilient network geared toward immune surveillance and genomic integrity. In contrast, CRC networks are densely wired around oncogenic drivers such as TP53, CCND1, and MYC, reflecting a complete rewiring toward proliferative dominance and immune escape (Fig. 9). These findings establish that disease progression in LS is not merely the loss of genomic fidelity, but a structural and functional collapse of protective regulatory architecture. They also suggest that miRNAs act as network‐level orchestrators rather than isolated effectors, making them ideal candidates for network‐informed biomarkers and therapeutic interventions [118, 119].

C‐LS and CRC may converge on overlapping risk genes. They diverge sharply in the functional pathways through which these genes are regulated and executed [120, 121]. The circulating miRNA profiles in LS (H‐LS and C‐LS) are enriched for immune, transcriptional, and signal relay pathways such as ImmuneSigDB, BioCarta, and PID, whereas CRC shows dominant enrichment in proliferation and stress‐adaptive circuits such as MAPK, Ras and Wnt signaling (Fig. 8A,B,D). This divergence indicates that the same genetic or phenotypic endpoints arise through distinct molecular mechanisms, shaped by underlying genomic context germline MMR deficiency in LS versus somatic accumulation in CRC [122, 123, 124]. This functional decoupling has critical implications. It means therapeutic targets and surveillance strategies cannot be generically applied across CRC subtypes. Instead, understanding how oncogenic pressure is molecularly channeled in each context enables the design of precision interventions. These findings emphasize the need to move beyond mutation presence toward a pathway‐centric understanding of risk and progression, particularly in the management of hereditary cancer syndromes.

The enrichment analysis reveals that circulating miRNAs dysregulated in CRC converge on highly conserved, canonical pathways central to tumor development. These include MAPK, Wnt, Ras signaling, as well as cellular senescence (Fig. 7). This convergence suggests that miRNA dysregulation in CRC is not stochastic but is systematically aligned with core developmental and oncogenic circuits that govern cell proliferation and differentiation [125]. Moreover, enrichment in processes like axonogenesis, GTPase signaling and chromatin remodeling reflects a reactivation of embryonic and neuronal programs, a hallmark of tumor plasticity and invasion [126]. These findings position circulating miRNAs as upstream regulators of transcriptional networks that rewire cell fate decisions thus highlighting their centrality in tumor biology [127, 128]. Importantly, the involvement of pathways linked to vesicle trafficking, synaptic architecture and transcription factor activity further imply that miRNAs not only regulate intracellular processes but also facilitate intercellular communication and niche remodeling [129]. Biologically, this illustrates how CRC hijacks fundamental developmental systems to promote malignant growth. Mechanistically, this also emphasizes the multi‐domain regulatory reach of miRNAs in cancer [130, 131]. These results substantiate the functional relevance of circulating miRNAs beyond diagnostics, identifying them as integrators of oncogenic signaling with potential for targeted disruption in therapeutic strategies.

C‐LS exhibits a globally dysregulated miRNA landscape that is both quantitatively broader and temporally earlier than that of CRC, suggesting a fundamentally accelerated molecular trajectory driven by germline mismatch repair deficiency. This dysregulation encompasses both upregulated and downregulated miRNA classes, implying a global reorganization of post‐transcriptional regulation rather than isolated pathway perturbations (Fig. 6). These data elevate miRNAs from passive biomarkers to potential indicators of molecular velocity capturing the pace and intensity of transformation in hereditary cancer. From a translational perspective, this argues for using circulating miRNAs not only for future early‐detection research, but also for dynamic risk stratification, enabling intensified molecular surveillance in LS variant heterozygotes with molecular signatures of accelerated progression. Such insights show the clinical urgency of integrating molecular biomarkers into hereditary cancer screening, where timing and trajectory rather than just the presence of risk, determine clinical outcomes.

The additional gene‐stratified analysis revealed that *MLH1, MSH2*, and *MSH6*‐associated LS patients harbor discrete circulating miRNA patterns. LS variant heterozygotes even in the absence of overt malignancy, exhibit a unique miRNAomic state that is molecularly distinct from cancer patients (Figs 2 and 4). This intermediate profile suggests that LS is not a binary state of health or disease but rather a dynamic, transitional condition [132]. The partial overlap in miRNA expression between H‐LS and C‐LS indicates a premalignant reprogramming trajectory that likely precedes histopathological transformation. Unlike CRC, where miRNA changes are abrupt and tumor‐driven, LS‐related shifts appear more subtle and anticipatory which reflects underlying germline MMR deficiencies interacting with age and immune cues as reflected in the prominence of ImmuneSigDB, BioCarta, and PID categories. These included signatures linked to cytokine signaling, antigen presentation, and transcription factor cascades such as NF‐κB and STAT pathways [133]. This transitional state provides a crucial biological window during which molecular surveillance and early interventions could be most effective. It also challenges current screening practices by revealing that tumor‐free LS variant heterozygotes already exhibit systemic molecular alterations, arguing for a paradigm shift from phenotype‐driven to molecular age‐risk driven surveillance. The existence of this liminal miRNAomic state underlines the potential of circulating miRNAs not only as biomarkers but as real‐time reporters of subclinical disease progression in CRC [134].

Age‐stratified miRNAs are not passive indicators of chronological aging but are active participants in shaping CRC susceptibility (Fig. 2) [68]. Several miRNAs exhibit dual roles serving both as biological sensors of systemic aging and as functional modulators of tumorigenesis through their regulation of the cell cycle, immune signaling and DNA repair pathways [68, 135]. The observation that specific miRNAs consistently track with age and simultaneously distinguish CRC from controls emphasizes their diagnostic value, particularly in the context of noninvasive, blood‐based biomarkers. Importantly, their age‐dependent expression patterns suggest they may influence the timing and trajectory of malignant transformation, potentially acting as rate‐limiting checkpoints that gate cellular transitions from homeostasis to dysplasia/dyskaryosis. This duality has major translational implications: it challenges the ‘one‐size‐fits‐all’ paradigm in biomarker development and emphasizes the need for age‐calibrated diagnostic molecular panels. Biologically, these findings imply that the miRNAome constitutes a temporally responsive layer of gene regulation that interfaces with intrinsic cancer risk, one that may be manipulated to delay or intercept oncogenic progression, especially in genetically predisposed populations such as those with LS [117].

Age in cancer biology is a central, biologically active determinant of miRNA regulatory architecture in CRC [136, 137]. The observation that miRNA profiles vary dramatically across age strata and that the vast majority of dysregulated miRNAs are age‐specific, suggests that aging fundamentally reprograms transcriptional control mechanisms, possibly through epigenetic drift, immune remodeling, or metabolic shifts (Fig. 1). This age‐dependent rewiring is not gradual but stratified, indicating that miRNAs act as temporal gatekeepers that either maintain tissue homeostasis or permit malignant transformation at critical life stages [138, 139]. In the context of LS, this age effect is particularly striking because it modulates miRNA expression even in genetically predisposed but phenotypically healthy individuals, implying a layered interaction between germline risk and biological aging [140, 141]. These results also imply that timing of molecular events rather than just their occurrence may influence cancer onset and progression and they argue for the development of age‐specific diagnostic thresholds and surveillance strategies. In broader terms, this positions the miRNAome as a dynamic system responsive to temporal physiological cues, redefining how we understand cancer risk trajectories in both hereditary and sporadic settings.

## Conclusions

This study provides a comprehensive age‐stratified and disease‐contextualized dissection of the circulating miRNAome in CRC and LS, addressing a critical gap in understanding how molecular risk evolves in LS compared to CRC according to age. By an integrated systems biology and bioinformatics approach, our results suggest that circulating miRNA profiles are not merely reflective of cancer presence but are dynamic biomarkers that capture premalignant reprogramming, immune erosion and oncogenic activation across different age groups and distinct clinical states. We have shown that LS variant heterozygotes especially those without overt cancer, exhibit a transitional miRNAomic architecture distinct from both healthy controls and CRC patients at different ages. This offers a new targeted molecular framework to detect early shifts in tumorigenic risk, particularly in early‐age onset cancers such as occurs in LS. Crucially, our study identifies specific age‐modulated miRNAs and regulatory hubs that serve dual roles as both biomarkers and functional effectors, mapping onto immunogenomics, DNA repair and developmental pathways. These new insights redefine the biology of LS as a process not only governed by germline mutations but also actively shaped by chronological age and transcriptional remodeling. Orthogonal validation in an independent primary colorectal tissue cohort supports external concordance of a prespecified six‐miRNA panel across biologically relevant mismatch repair‐related disease axes. Translationally, this work lays the foundation for age‐based miRNA guided molecular surveillance algorithms tailored to LS variant heterozygotes, enabling future early‐detection research before the histopathological transformation of cancer. Furthermore, the discovery of a distinct ‘oncogenic LS signature’ creates opportunities for noninvasive risk prediction and targeted intervention in LS. In summary, this study advances a new paradigm in cancer precision oncology where age, genetic predisposition and molecular dynamics converge to inform individualized molecular screening, prognosis, and prevention strategies.

## Conflict of interest

The authors declare no competing interests.

## Author contributions

RC conceived and designed the project, curated and analyzed the data, prepared the visualizations, and drafted the manuscript. MSUI contributed to data curation, formal analysis and visualization. RMX, RR, XL, and SS contributed to interpretation of the data and critically revised the manuscript. RC and SS supervised the project. All authors reviewed and approved the final manuscript.

## Supporting information


**Figure S1.** Circulating miRNA interaction network.
**Figure S2**. Z‐score heatmap of the top 10 miRNAs ranked by nominal *P* value in pooled cancer versus healthy participants.
**Figure S3**. Hierarchical heatmap of miRNA expression in participants aged 50 years or younger.
**Figure S4**. Hierarchical heatmap of miRNA expression in participants aged > 50 years.
**Figure S5**. Hierarchical heatmap of miRNA expression by age group (≤ 50 versus > 50 years).
**Figure S6**. Nominal *P* values from age, clinical‐group and age‐group miRNA association analyses.
**Figure S7**. MA plot of differential miRNA expression.
**Figure S8**. Volcano plot of nominal differential miRNA expression in pooled cancer versus healthy participants.
**Figure S9**. Dispersion estimates for the miRNA differential‐expression model.
**Figure S10**. Volcano plot of differential miRNA expression in participants aged > 50 versus ≤ 50 years.
**Figure S11**. Volcano plot of differential miRNA expression in male versus female participants.
**Figure S12**. Volcano plot of differential miRNA expression in CRC versus healthy participants.
**Figure S13**. Volcano plot of differential miRNA expression in Lynch versus non‐Lynch participants.
**Figure S14**. Volcano plot of differential miRNA expression in H‐LS versus C‐LS.
**Figure S15**. Volcano plot of differential miRNA expression in H‐LS versus CRC.
**Figure S16**. Annotated volcano plot of nominal differential miRNA expression in pooled cancer versus healthy participants.
**Figure S17**. Volcano plot of differential miRNA expression in H‐LS versus CRC.
**Figure S18**. Volcano plot of differential miRNA expression in H‐LS versus C‐LS.
**Figure S19**. Volcano plot of differential miRNA expression in CTRL versus CRC.
**Figure S20**. Volcano plot of differential miRNA expression in CTRL versus C‐LS.
**Figure S21**. Annotated volcano plot of differential miRNA expression in CTRL versus CRC.
**Figure S22**. Annotated volcano plot of differential miRNA expression in CTRL versus C‐LS.

**Table S1**. Demographic and clinical metadata for the 155 discovery‐cohort samples.
**Table S2**. Normalized expression matrix for 228 filtered circulating miRNAs across 155 samples.
**Table S3**. Filtered circulating‐miRNA read‐count matrix across 155 samples.
**Table S4**. Age‐association regression results for circulating miRNAs.
**Table S5**. Cancer‐condition‐association regression results for circulating miRNAs.
**Table S6**. Differential miRNA‐expression results for CTRL versus C‐LS.
**Table S7**. Differential miRNA‐expression results for CTRL versus CRC.
**Table S8**. Differential miRNA‐expression results for H‐LS versus C‐LS.
**Table S9**. Differential miRNA‐expression results for H‐LS versus CRC.
**Table S10**. Circulating‐miRNA count matrix for CTRL and CRC samples.
**Table S11**. Circulating‐miRNA count matrix for H‐LS and C‐LS samples.
**Table S12**. Circulating‐miRNA count matrix for H‐LS and CRC samples.
**Table S13**. Circulating‐miRNA count matrix for CTRL and C‐LS samples.
**Table S14**. Top hub miRNAs identified by coexpression‐network analysis.
**Table S15**. Coexpression‐module assignments for filtered circulating miRNAs.
**Table S16**. Correlations between miRNA module eigengenes and age, sex and disease‐status traits.
**Table S17**. *P* values for correlations between miRNA module eigengenes and age, sex and disease‐status traits.
**Table S18**. Correlation coefficients between circulating‐miRNA expression and age in the healthy subgroup.
**Table S19**. Correlation coefficients between circulating‐miRNA expression and age in the cancer subgroup.
**Table S20**. Linear‐regression results for circulating‐miRNA expression by age and CRC/CTRL status.
**Table S21**. Differential miRNA‐expression results for H‐LS versus C‐LS.
**Table S22**. Differential miRNA‐expression results for H‐LS versus CRC.
**Table S23**. Differential miRNA‐expression results for CTRL versus CRC.
**Table S24**. Differential miRNA‐expression results for CTRL versus C‐LS.
**Table S25**. Target genes of downregulated miRNAs in C‐LS.
**Table S26**. Target genes of downregulated miRNAs in CRC.
**Table S27**. Predicted target‐interaction map for downregulated miRNAs in C‐LS.
**Table S28**. Predicted target‐interaction map for downregulated miRNAs in CRC.
**Table S29**. Predicted miRNA‐target interaction map for CTRL versus CRC.
**Table S30**. Predicted miRNA‐target interaction map for H‐LS versus C‐LS.
**Table S31**. Predicted target‐interaction map for upregulated miRNAs in C‐LS.
**Table S32**. Predicted target‐interaction map for upregulated miRNAs in CRC.
**Table S33**. Circulating‐miRNA expression matrix for age‐stratified UMAP analysis.
**Table S34**. Circulating‐miRNA expression matrix for disease‐group UMAP analysis.
**Table S35**. External validation dataset for six pre‐specified miRNAs in GSE30454.

## Data Availability

This secondary analysis used publicly available, de‐identified data. Discovery cohort small‐RNA sequencing data and metadata are available from the NCBI Gene Expression Omnibus under accession GSE198834, and the external primary colorectal tissue validation data are available under accession GSE30454. No new biological samples, materials, or reagents were generated.
