# Supplementary material for: Unique circulating miRNAome signatures captured with distinct molecular states in Lynch syndrome and sporadic colorectal cancer
Source: Mol Oncol. 2026 Oct 7:10.1002/1878-0261.70338. Online ahead of print. doi: 10.1002/1878-0261.70338 (PMC13643886; doi:10.1002/1878-0261.70338)
Supplement: Supplementary file 1 [file MOL2-9999-0-s001.zip › Figure_S13.pdf]

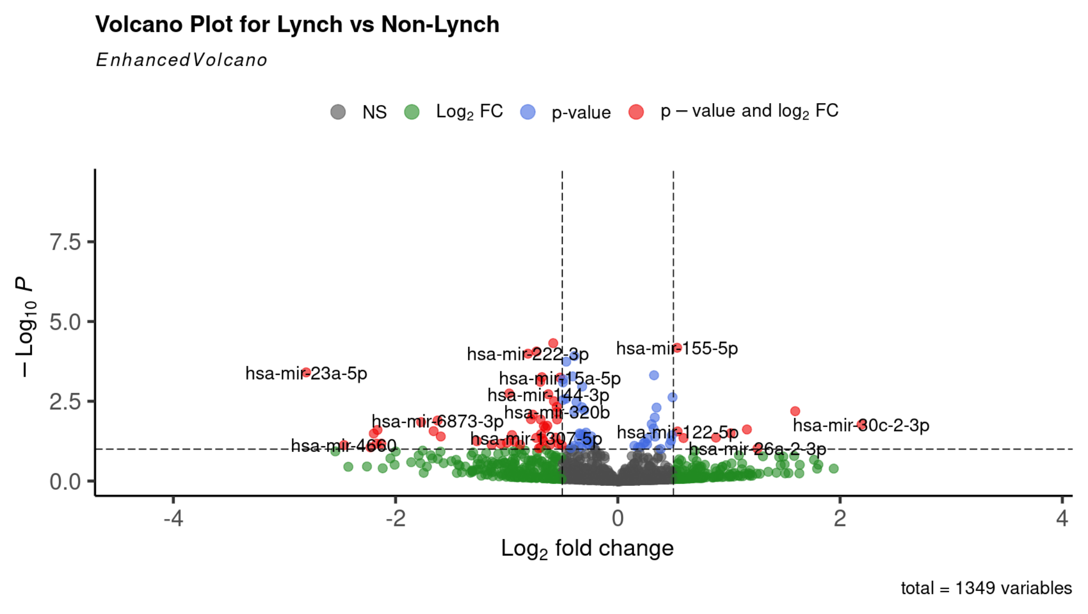

Volcano Plot for Lynch vs Non-Lynch
EnhancedVolcano
NS
Log2 FC
p-value
p – value and log2 FC
– Log10 P
7.5
5.0
2.5
0.0
-4
-2
0
2
4
Log2 fold change
hsa-mir-23a-5p
hsa-mir-222-3p
hsa-mir-155-5p
hsa-mir-15a-5p
hsa-mir-144-3p
hsa-mir-320b
hsa-mir-6873-3p
hsa-mir-122-5p
hsa-mir-30c-2-3p
hsa-mir-1307-5p
hsa-mir-4660
hsa-mir-26a-2-3p
total = 1349 variables
