# Supplementary material for: Unique circulating miRNAome signatures captured with distinct molecular states in Lynch syndrome and sporadic colorectal cancer
Source: Mol Oncol. 2026 Oct 7:10.1002/1878-0261.70338. Online ahead of print. doi: 10.1002/1878-0261.70338 (PMC13643886; doi:10.1002/1878-0261.70338)
Supplement: Supplementary file 1 [file MOL2-9999-0-s001.zip › Figure_S17.pdf]

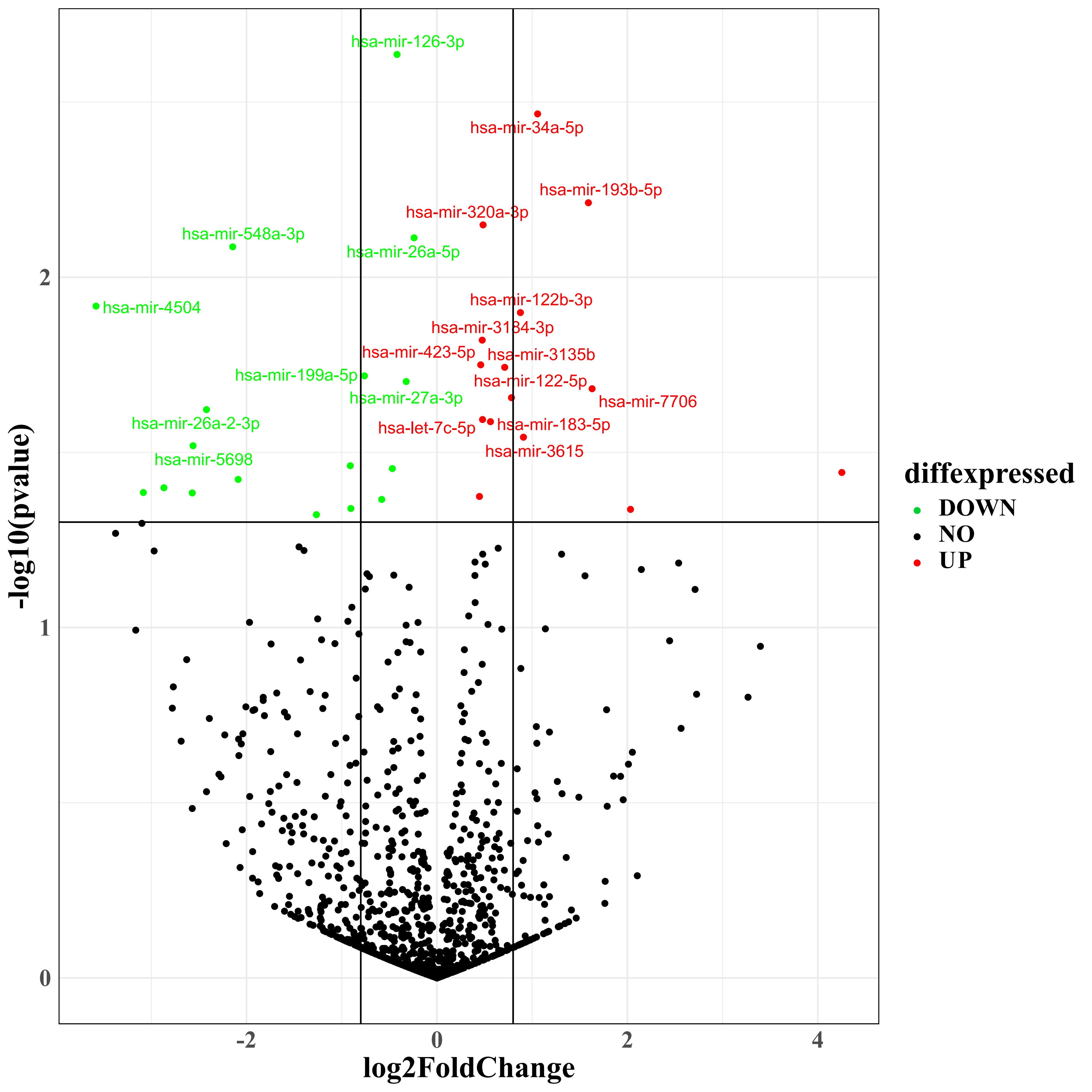

hsa-mir-126-3p
hsa-mir-34a-5p
hsa-mir-193b-5p
hsa-mir-320a-3p
hsa-mir-548a-3p
hsa-mir-26a-5p
hsa-mir-4504
hsa-mir-122b-3p
hsa-mir-3184-3p
hsa-mir-423-5p
hsa-mir-3135b
hsa-mir-199a-5p
hsa-mir-122-5p
hsa-mir-7706
hsa-mir-27a-3p
hsa-mir-26a-2-3p
hsa-let-7c-5p
hsa-mir-183-5p
hsa-mir-3615
hsa-mir-5698
-log10(pvalue)
2
1
0
-2
0
2
4
log2FoldChange
diffexpressed
DOWN
NO
UP
