# Supplementary material for: Unique circulating miRNAome signatures captured with distinct molecular states in Lynch syndrome and sporadic colorectal cancer
Source: Mol Oncol. 2026 Oct 7:10.1002/1878-0261.70338. Online ahead of print. doi: 10.1002/1878-0261.70338 (PMC13643886; doi:10.1002/1878-0261.70338)
Supplement: Supplementary file 1 [file MOL2-9999-0-s001.zip › Figure_S18.pdf]

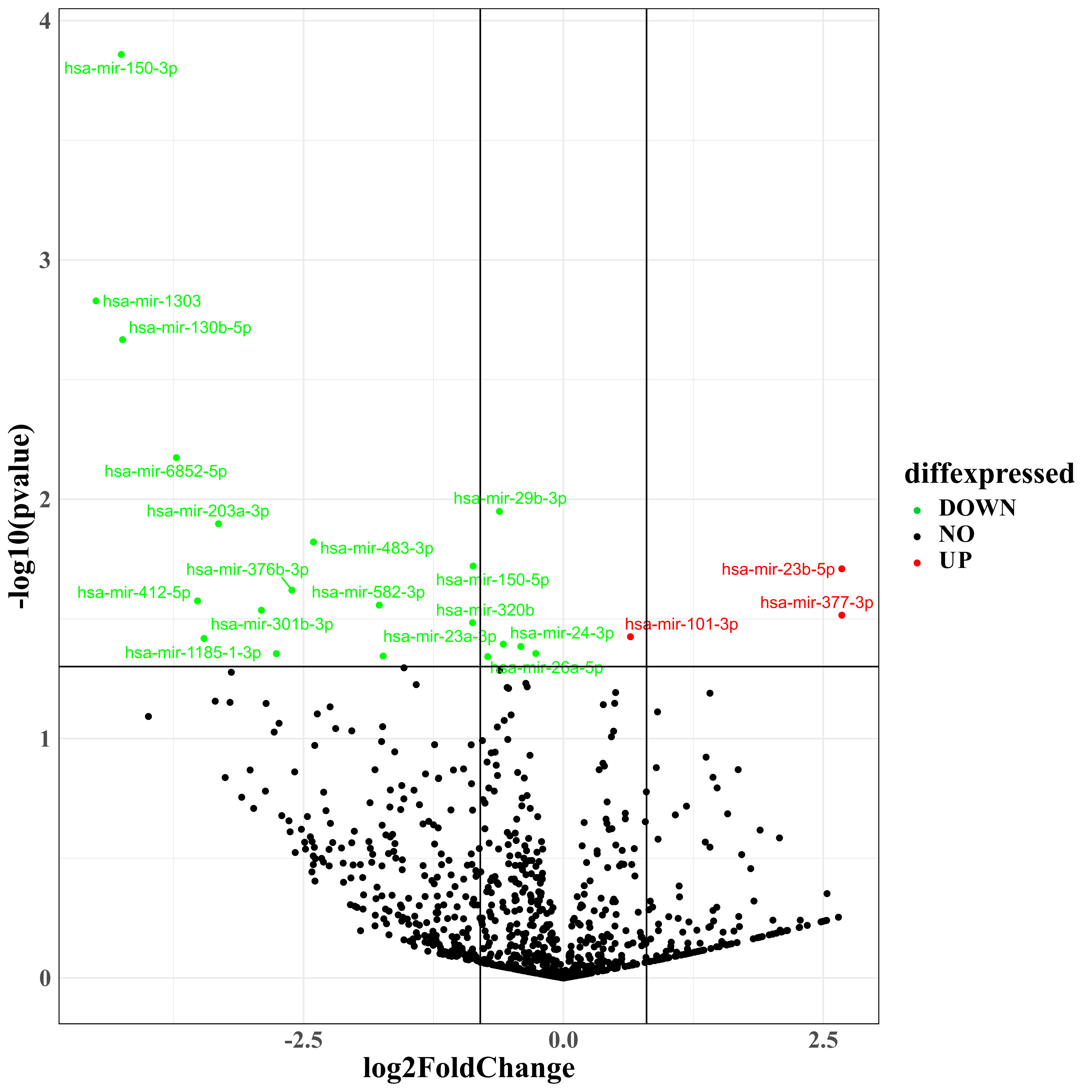

hsa-mir-150-3p
hsa-mir-1303
hsa-mir-130b-5p
hsa-mir-6852-5p
hsa-mir-29b-3p
hsa-mir-203a-3p
hsa-mir-483-3p
hsa-mir-376b-3p
hsa-mir-150-5p
hsa-mir-412-5p
hsa-mir-582-3p
hsa-mir-320b
hsa-mir-301b-3p
hsa-mir-23a-3p
hsa-mir-24-3p
hsa-mir-1185-1-3p
hsa-mir-26a-5p
hsa-mir-23b-5p
hsa-mir-377-3p
hsa-mir-101-3p
diffexpressed
DOWN
NO
UP
-log10(pvalue)
log2FoldChange
0
1
2
3
4
-2.5
0.0
2.5
