# Supplementary material for: Unique circulating miRNAome signatures captured with distinct molecular states in Lynch syndrome and sporadic colorectal cancer
Source: Mol Oncol. 2026 Oct 7:10.1002/1878-0261.70338. Online ahead of print. doi: 10.1002/1878-0261.70338 (PMC13643886; doi:10.1002/1878-0261.70338)
Supplement: Supplementary file 1 [file MOL2-9999-0-s001.zip › Figure_S21.pdf]

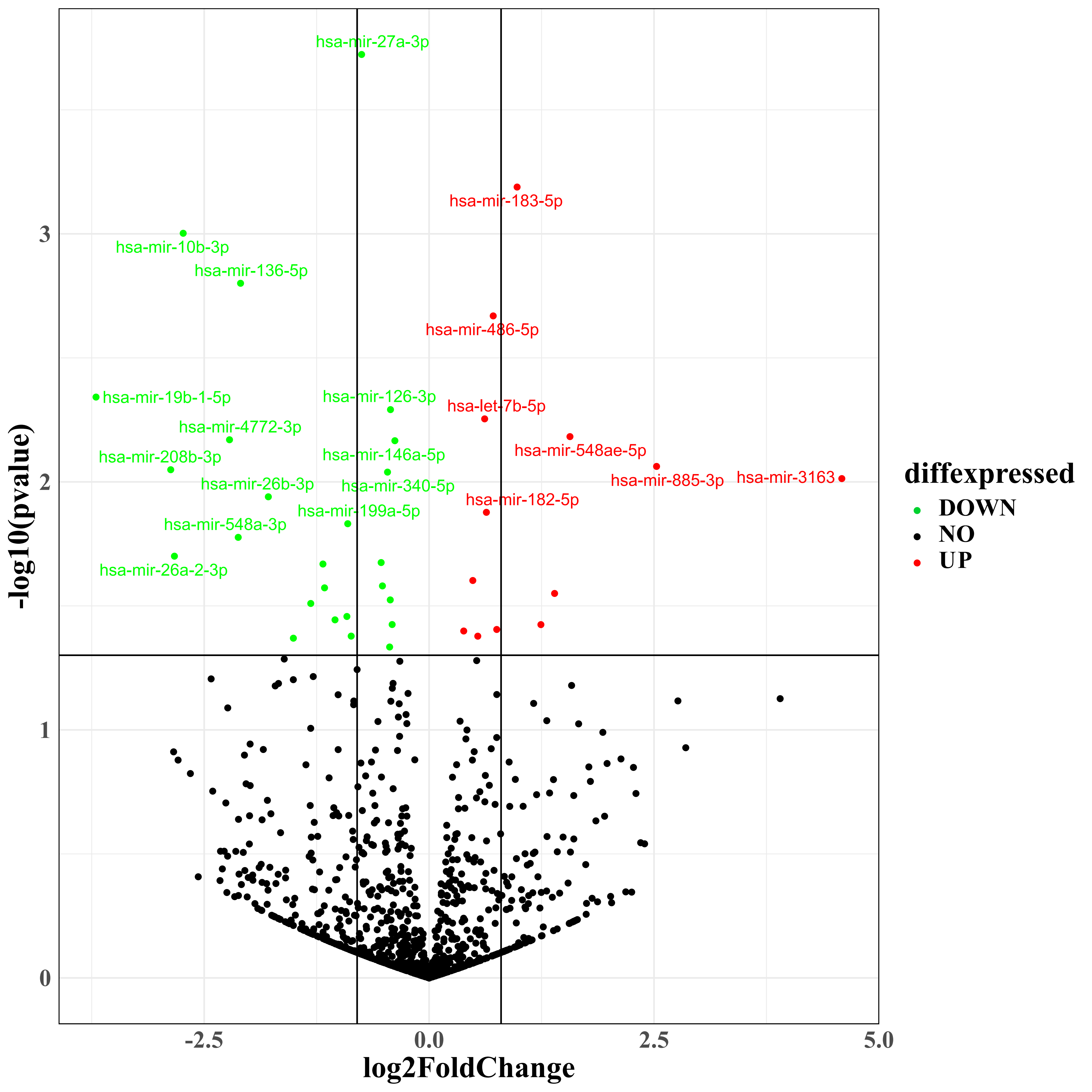

hsa-mir-27a-3p
hsa-mir-183-5p
hsa-mir-10b-3p
hsa-mir-136-5p
hsa-mir-486-5p
hsa-mir-19b-1-5p
hsa-mir-126-3p
hsa-let-7b-5p
hsa-mir-4772-3p
hsa-mir-146a-5p
hsa-mir-548ae-5p
hsa-mir-208b-3p
hsa-mir-885-3p
hsa-mir-3163
hsa-mir-26b-3p
hsa-mir-340-5p
hsa-mir-182-5p
hsa-mir-199a-5p
hsa-mir-548a-3p
hsa-mir-26a-2-3p
-log10(pvalue)
log2FoldChange
0
1
2
3
-2.5
0.0
2.5
5.0
diffexpressed
DOWN
NO
UP
