# Supplementary material for: Unique circulating miRNAome signatures captured with distinct molecular states in Lynch syndrome and sporadic colorectal cancer
Source: Mol Oncol. 2026 Oct 7:10.1002/1878-0261.70338. Online ahead of print. doi: 10.1002/1878-0261.70338 (PMC13643886; doi:10.1002/1878-0261.70338)
Supplement: Supplementary file 1 [file MOL2-9999-0-s001.zip › Figure_S22.pdf]

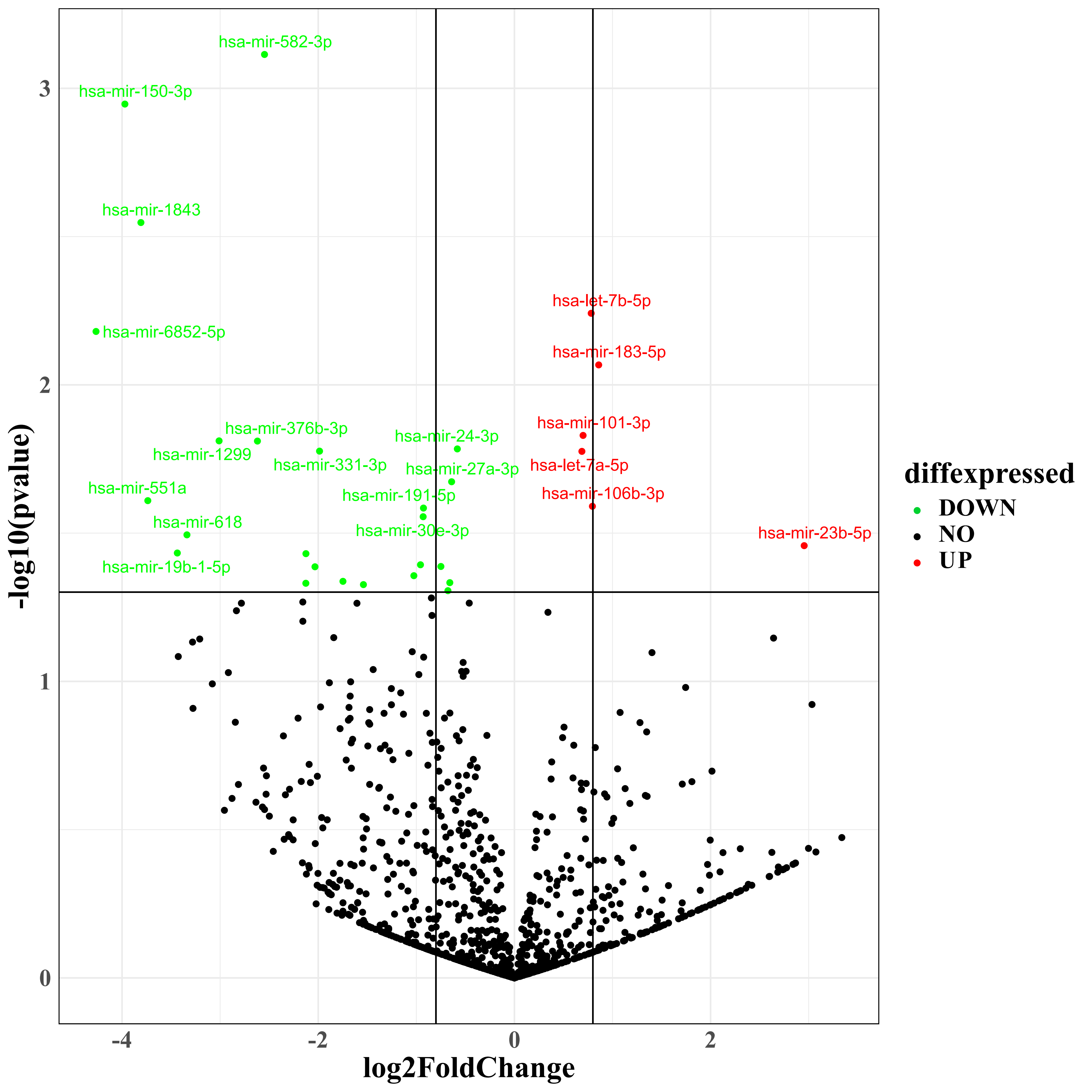
hsa-mir-582-3p
hsa-mir-150-3p
hsa-mir-1843
hsa-mir-6852-5p
hsa-mir-376b-3p
hsa-mir-1299
hsa-mir-331-3p
hsa-mir-24-3p
hsa-mir-27a-3p
hsa-mir-551a
hsa-mir-191-5p
hsa-mir-618
hsa-mir-30e-3p
hsa-mir-19b-1-5p
hsa-let-7b-5p
hsa-mir-183-5p
hsa-mir-101-3p
hsa-let-7a-5p
hsa-mir-106b-3p
hsa-mir-23b-5p
-log10(pvalue)
0
1
2
3
log2FoldChange
-4
-2
0
2
diffexpressed
DOWN
NO
UP
