# Supplementary material for: Unique circulating miRNAome signatures captured with distinct molecular states in Lynch syndrome and sporadic colorectal cancer
Source: Mol Oncol. 2026 Oct 7:10.1002/1878-0261.70338. Online ahead of print. doi: 10.1002/1878-0261.70338 (PMC13643886; doi:10.1002/1878-0261.70338)
Supplement: Supplementary file 1 [file MOL2-9999-0-s001.zip › Supplementary_Figure_Legends.docx]

**Supplementary Figure Legends**

**Title: Unique circulating miRNAome signatures captured with distinct molecular states in Lynch syndrome and sporadic colorectal cancer**

**Supplementary Figure S1.** **Circulating miRNA interaction network**. Top upregulated discovery miRNAs were submitted to miRNet, and the source workflow filtered interactions using high-confidence conserved predictions from TargetScan Human 8.0 and strongly supported experimentally validated interactions from miRTarBase 9.0 before visualization in Cytoscape. Circular nodes are miRNAs and connecting lines are the associations retained in the exported network. The artwork highlights let-7i and let-7g* in red and shows the other miRNAs in grey; the source package does not provide a biological key for this colour emphasis, so red should not be interpreted as a formal hub category. miRNA, microRNA.

**Supplementary Figure S2.** **Z-score heatmap of the 10 miRNAs with the smallest nominal P values in the pooled cancer-versus-healthy discovery contrast.** Cancer combined C-LS and CRC participants (n = 37), and healthy combined H-LS and CTRL participants (n = 118). Rows are miRNAs, columns are participants and colour represents the standardized score used in the source workflow. The ranking is nominal: 1 of 228 tested miRNAs met a Benjamini-Hochberg false-discovery-rate threshold of 0.05. C-LS, cancer-associated Lynch syndrome; CRC, sporadic colorectal cancer; CTRL, healthy control; H-LS, non-malignant Lynch syndrome; miRNA, microRNA.

**Supplementary Figure S3.** **Hierarchical heatmap of the 228 filtered circulating miRNAs in participants aged 50 years or younger (n = 39)**. Rows are miRNAs, columns are participants and colour represents the standardized expression value used in the source workflow. Dendrograms show unsupervised hierarchical clustering of rows and columns. miRNA, microRNA.

**Supplementary Figure S4.** **Hierarchical heatmap of the 228 filtered circulating miRNAs** in participants older than 50 years (n = 116). Rows are miRNAs, columns are participants and colour represents the standardized expression value used in the source workflow. Dendrograms show unsupervised hierarchical clustering of rows and columns. miRNA, microRNA.

**Supplementary Figure S5**. **Hierarchical heatmap of the 228 filtered circulating miRNAs in all 155 participants,** annotated as 50 years or younger (n = 39) or older than 50 years (n = 116). Rows are miRNAs, columns are participants and colour represents the standardized expression value used in the source workflow. The row dendrogram shows unsupervised hierarchical clustering of miRNAs. miRNA, microRNA.

**Supplementary Figure S6.** **Nominal P values from three circulating-miRNA association analyses.** From left to right, panels show linear regression of expression on continuous age; one-way analysis of variance across CTRL, H-LS, C-LS and CRC; and one-way analysis of variance across Young (<51 years), Middle (51-64 years) and Older (>64 years) groups. Each panel shows the 50 miRNAs with the smallest unadjusted P values, ordered alphabetically; lines connect points for display only and the panels use different y-axis scales. C-LS, cancer-associated Lynch syndrome; CRC, sporadic colorectal cancer; CTRL, healthy control; H-LS, non-malignant Lynch syndrome; miRNA, microRNA.

**Supplementary Figure S7.** **Mean-average (MA) plot from the DESeq2 pooled cancer-versus-healthy model**, comprising 228 filtered miRNAs. Cancer combined C-LS and CRC participants (n = 37), and healthy combined H-LS and CTRL participants (n = 118). Each point is one miRNA; the x-axis is mean normalized abundance and the y-axis is log2 fold change, with positive values indicating higher expression in pooled cancer. Red marks the 18 miRNAs with unadjusted P < 0.05, whereas grey denotes P >= 0.05; one miRNA met Benjamini-Hochberg false-discovery-rate < 0.05. Dashed lines at log2 fold change = -2 and 2 are reference lines. C-LS, cancer-associated Lynch syndrome; CRC, sporadic colorectal cancer; CTRL, healthy control; DESeq2, Differential Expression Sequencing 2 R package; H-LS, non-malignant Lynch syndrome; miRNA, microRNA.

**Supplementary Figure S8.** **Volcano plot from the DESeq2 pooled cancer-versus-healthy model**, comprising 228 filtered miRNAs. Cancer combined C-LS and CRC participants (n = 37), and healthy combined H-LS and CTRL participants (n = 118). The x-axis is log2 fold change and the y-axis is -log10 of the unadjusted P value; positive change indicates higher expression in pooled cancer. At nominal P < 0.05 without a fold-change cut-off, 14 miRNAs were higher (red) and 4 lower (purple); 210 were not significant (grey). One miRNA met Benjamini-Hochberg false-discovery-rate < 0.05. C-LS, cancer-associated Lynch syndrome; CRC, sporadic colorectal cancer; CTRL, healthy control; DESeq2, Differential Expression Sequencing 2 R package; H-LS, non-malignant Lynch syndrome; miRNA, microRNA.

**Supplementary Figure S9.** **Dispersion estimates from the DESeq2 negative-binomial pooled cancer-versus-healthy model** for 228 filtered miRNAs. Black points are gene-wise estimates, the red curve is the fitted mean-dispersion trend and blue points are the final estimates used for inference. Cancer combined C-LS and CRC participants (n = 37), and healthy combined H-LS and CTRL participants (n = 118). C-LS, cancer-associated Lynch syndrome; CRC, sporadic colorectal cancer; CTRL, healthy control; DESeq2, Differential Expression Sequencing 2 R package; H-LS, non-malignant Lynch syndrome; miRNA, microRNA.

**Supplementary Figure S10.** **Volcano plot from the DESeq2 contrast of participants** older than 50 years (n = 116) versus those aged 50 years or younger (n = 39). Each point is one miRNA; the x-axis is log2 fold change and the y-axis is -log10 of the unadjusted P value. Positive change indicates higher expression in the older group. Coloured points meet both unadjusted P < 0.10 and absolute log2 fold change >= 0.5; labelled points are selected miRNAs. DESeq2, Differential Expression Sequencing 2 R package; miRNA, microRNA.

**Supplementary Figure S11.** **Volcano plot from the DESeq2 contrast of male (n = 78) versus female participants (n = 77).** Each point is one miRNA; the x-axis is log2 fold change and the y-axis is -log10 of the unadjusted P value. Positive change indicates higher expression in male participants. Coloured points meet both unadjusted P < 0.10 and absolute log2 fold change >= 0.5; labelled points are selected miRNAs. DESeq2, Differential Expression Sequencing 2 R package; miRNA, microRNA.

**Supplementary Figure S12.** **Volcano plot from the DESeq2 contrast of pooled cancer (C-LS plus CRC; n = 37) versus pooled healthy participants (H-LS plus CTRL; n = 118).** Each point is one miRNA; the x-axis is log2 fold change and the y-axis is -log10 of the unadjusted P value. Positive change indicates higher expression in pooled cancer. Coloured points meet both unadjusted P < 0.10 and absolute log2 fold change >= 0.5; labelled points are selected miRNAs. C-LS, cancer-associated Lynch syndrome; CRC, sporadic colorectal cancer; CTRL, healthy control; DESeq2, Differential Expression Sequencing 2 R package; H-LS, non-malignant Lynch syndrome; miRNA, microRNA.

**Supplementary Figure S13.** **Volcano plot from the DESeq2 contrast of Lynch-syndrome participants (H-LS plus C-LS; n = 94) versus non-Lynch participants (CTRL plus CRC; n = 61).** Each point is one miRNA; the x-axis is log2 fold change and the y-axis is -log10 of the unadjusted P value. Positive change indicates higher expression in the pooled Lynch-syndrome group. Coloured points meet both unadjusted P < 0.10 and absolute log2 fold change >= 0.5; labelled points are selected miRNAs. C-LS, cancer-associated Lynch syndrome; CRC, sporadic colorectal cancer; CTRL, healthy control; DESeq2, Differential Expression Sequencing 2 R package; H-LS, non-malignant Lynch syndrome; miRNA, microRNA.

**Supplementary Figure S14.** **Volcano plot from the DESeq2 treatment-only contrast of H-LS (n = 81) and C-LS (n = 13).** Positive log2 fold change indicates higher expression in C-LS. Red and blue points meet unadjusted P < 0.05 and absolute log2 fold change > 0.1 in the positive and negative directions, respectively; grey points do not. Dashed reference lines mark P = 0.05 and absolute log2 fold change = 0.8, and labels identify the 20 smallest unadjusted P values. No miRNA met Benjamini-Hochberg false-discovery-rate < 0.05. C-LS, cancer-associated Lynch syndrome; DESeq2, Differential Expression Sequencing 2 R package; H-LS, non-malignant Lynch syndrome; miRNA, microRNA.

**Supplementary Figure S15.** **Volcano plot from the DESeq2 treatment-only contrast of H-LS (n = 81) and CRC (n = 24).** Positive log2 fold change indicates higher expression in CRC. Red and blue points meet unadjusted P < 0.05 and absolute log2 fold change > 0.1 in the positive and negative directions, respectively; grey points do not. Dashed reference lines mark P = 0.05 and absolute log2 fold change = 0.8, and labels identify the 20 smallest unadjusted P values. No miRNA met Benjamini-Hochberg false-discovery-rate < 0.05. CRC, sporadic colorectal cancer; DESeq2, Differential Expression Sequencing 2 R package; H-LS, non-malignant Lynch syndrome; miRNA, microRNA.

**Supplementary Figure S16.** **Alternate annotated export of the pooled cancer-versus-healthy DESeq2 volcano plot shown in Supplementary Figure S8.** The contrast, axes, sample sizes and nominal significance categories are identical to Supplementary Figure S8; this export changes the selected miRNA annotations. One of 228 miRNAs met Benjamini-Hochberg false-discovery-rate < 0.05. C-LS, cancer-associated Lynch syndrome; CRC, sporadic colorectal cancer; CTRL, healthy control; DESeq2, Differential Expression Sequencing 2 R package; H-LS, non-malignant Lynch syndrome; miRNA, microRNA.

**Supplementary Figure S17.** **Alternate export of the H-LS-versus-CRC DESeq2 contrast shown in Supplementary Figure S15**. Positive log2 fold change indicates higher expression in CRC. Colours, reference lines and annotation criteria are identical to Supplementary Figure S15, and no miRNA met Benjamini-Hochberg false-discovery-rate < 0.05. CRC, sporadic colorectal cancer; DESeq2, Differential Expression Sequencing 2 R package; H-LS, non-malignant Lynch syndrome; miRNA, microRNA.

**Supplementary Figure S18.** **Alternate export of the H-LS-versus-C-LS DESeq2 contrast shown in Supplementary Figure S14.** Positive log2 fold change indicates higher expression in C-LS. Colours, reference lines and annotation criteria are identical to Supplementary Figure S14, and no miRNA met Benjamini-Hochberg false-discovery-rate < 0.05. C-LS, cancer-associated Lynch syndrome; DESeq2, Differential Expression Sequencing 2 R package; H-LS, non-malignant Lynch syndrome; miRNA, microRNA.

**Supplementary Figure S19.** **Volcano plot from the DESeq2 treatment-only contrast of CTRL (n = 37) and CRC (n = 24).** Positive log2 fold change indicates higher expression in CRC. Red and blue points meet unadjusted P < 0.05 and absolute log2 fold change > 0.1 in the positive and negative directions, respectively; grey points do not. Dashed reference lines mark P = 0.05 and absolute log2 fold change = 0.8, and labels identify the 20 smallest unadjusted P values. No miRNA met Benjamini-Hochberg false-discovery-rate < 0.05. CRC, sporadic colorectal cancer; CTRL, healthy control; DESeq2, Differential Expression Sequencing 2 R package; miRNA, microRNA.

**Supplementary Figure S20.** **Volcano plot from the DESeq2 treatment-only contrast of CTRL (n = 37) and C-LS (n = 13).** Positive log2 fold change indicates higher expression in C-LS. Red and blue points meet unadjusted P < 0.05 and absolute log2 fold change > 0.1 in the positive and negative directions, respectively; grey points do not. Dashed reference lines mark P = 0.05 and absolute log2 fold change = 0.8, and labels identify the 20 smallest unadjusted P values. No miRNA met Benjamini-Hochberg false-discovery-rate < 0.05. C-LS, cancer-associated Lynch syndrome; CTRL, healthy control; DESeq2, Differential Expression Sequencing 2 R package; miRNA, microRNA.

**Supplementary Figure S21. Alternate export of the CTRL-versus-CRC DESeq2 contrast shown in Supplementary Figure S19.** Positive log2 fold change indicates higher expression in CRC. Colours, reference lines and annotation criteria are identical to Supplementary Figure S19, and no miRNA met Benjamini-Hochberg false-discovery-rate < 0.05. CRC, sporadic colorectal cancer; CTRL, healthy control; DESeq2, Differential Expression Sequencing 2 R package; miRNA, microRNA.

**Supplementary Figure S22.** **Alternate export of the CTRL-versus-C-LS DESeq2 contrast shown in Supplementary Figure S20.** Positive log2 fold change indicates higher expression in C-LS. Colours, reference lines and annotation criteria are identical to Supplementary Figure S20, and no miRNA met Benjamini-Hochberg false-discovery-rate < 0.05. C-LS, cancer-associated Lynch syndrome; CTRL, healthy control; DESeq2, Differential Expression Sequencing 2 R package; miRNA, microRNA.
