# Supplementary material for: Unique circulating miRNAome signatures captured with distinct molecular states in Lynch syndrome and sporadic colorectal cancer
Source: Mol Oncol. 2026 Oct 7:10.1002/1878-0261.70338. Online ahead of print. doi: 10.1002/1878-0261.70338 (PMC13643886; doi:10.1002/1878-0261.70338)
Supplement: Supplementary file 1 [file MOL2-9999-0-s001.zip › Figure_S1.pdf]

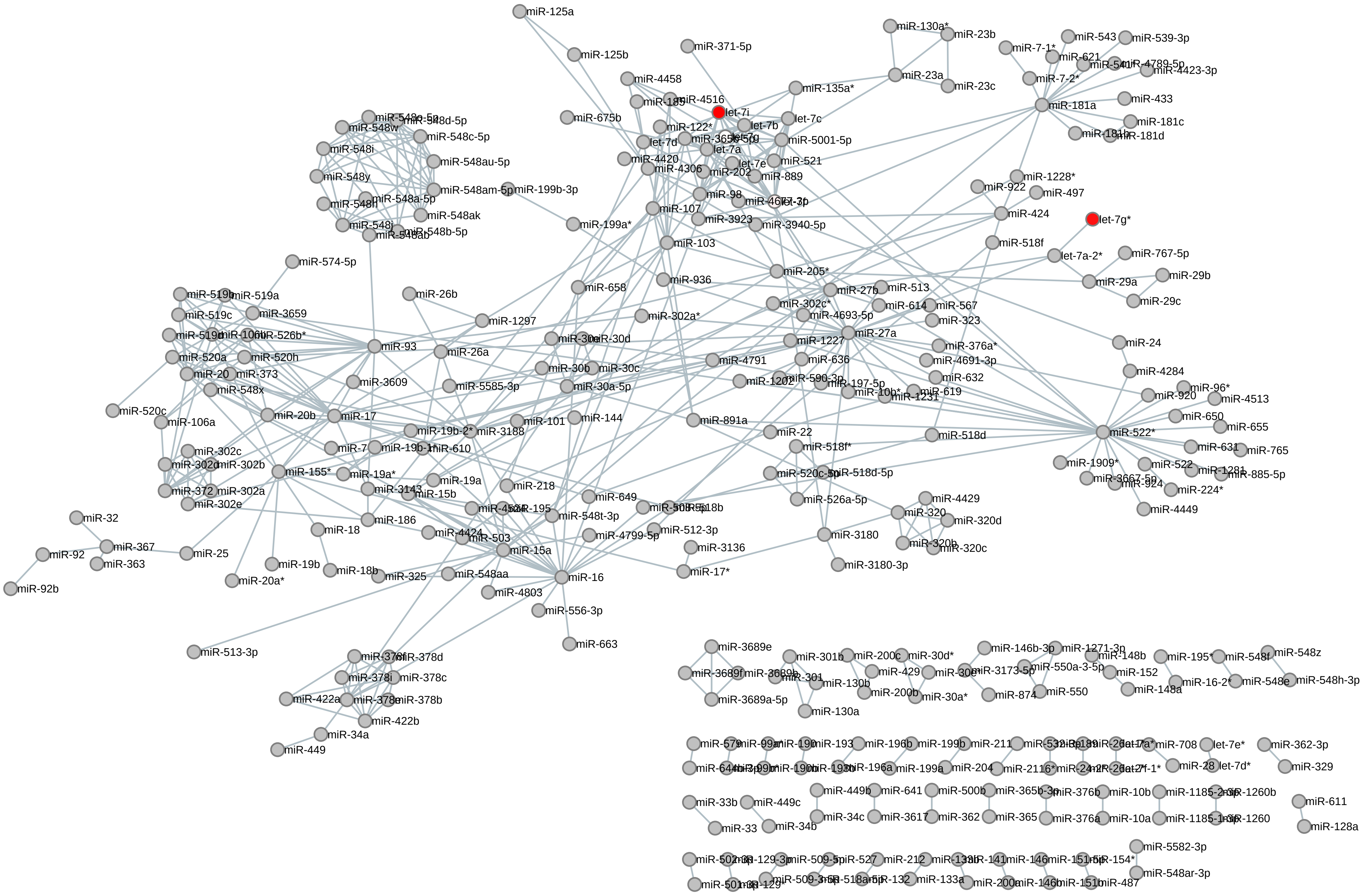

miR-125a
miR-125b
miR-371-5p
miR-130a*
miR-23b
miR-23a
miR-23c
miR-7-1*
miR-543
miR-539-3p
miR-621
miR-7-2*
miR-4423-3p
miR-181a
miR-433
miR-181c
miR-181d
miR-4458
miR-4516
let-7i
miR-135a*
let-7c
let-7b
miR-675b
miR-122*
let-7d
let-7a
miR-5001-5p
miR-521
let-7e
miR-4420
miR-4306
miR-202
miR-889
miR-98
miR-107
miR-3923
miR-3940-5p
miR-103
miR-1228*
miR-922
miR-497
miR-424
let-7g*
miR-518f
let-7a-2*
miR-767-5p
miR-29a
miR-29b
miR-29c
miR-205*
miR-936
miR-658
miR-27b
miR-513
miR-614
miR-567
miR-302c*
miR-4693-5p
miR-323
miR-27a
miR-1227
miR-376a*
miR-636
miR-4691-3p
miR-632
miR-24
miR-4284
miR-548w
miR-548d-5p
miR-548c-5p
miR-548i
miR-548au-5p
miR-548y
miR-548am-5p
miR-548a-5p
miR-548h
miR-548ak
miR-548j
miR-548b-5p
miR-548ab
miR-199b-3p
miR-199a*
miR-574-5p
miR-26b
miR-519b
miR-519a
miR-3659
miR-519c
miR-526b*
miR-1297
miR-302a*
miR-93
miR-26a
miR-30d
miR-520a
miR-520h
miR-30b
miR-30c
miR-4791
miR-20
miR-373
miR-3609
miR-5585-3p
miR-30a-5p
miR-1202
miR-197-5p
miR-10b*
miR-1231
miR-619
miR-920
miR-96*
miR-4513
miR-520c
miR-548x
miR-106a
miR-20b
miR-17
miR-101
miR-144
miR-891a
miR-650
miR-655
miR-19b-2*
miR-3188
miR-22
miR-518d
miR-522*
miR-302c
miR-610
miR-518f*
miR-631
miR-765
miR-1909*
miR-522
miR-1281
miR-885-5p
miR-302d
miR-302b
miR-155*
miR-19a*
miR-19a
miR-218
miR-520c-5p
miR-518d-5p
miR-3667-5p
miR-924
miR-224*
miR-372
miR-302a
miR-3143
miR-15b
miR-649
miR-526a-5p
miR-4429
miR-4449
miR-302e
miR-32
miR-186
miR-548t-3p
miR-320
miR-320d
miR-367
miR-18
miR-4424
miR-503
miR-4799-5p
miR-512-3p
miR-3180
miR-320b
miR-320c
miR-92
miR-363
miR-25
miR-15a
miR-3136
miR-19b
miR-18b
miR-325
miR-548aa
miR-16
miR-17*
miR-3180-3p
miR-92b
miR-20a*
miR-4803
miR-556-3p
miR-663
miR-513-3p
miR-3689e
miR-301b
miR-200c
miR-30d*
miR-146b-3p
miR-1271-3p
miR-148b
miR-195*
miR-548f
miR-548z
miR-3689f
miR-301
miR-429
miR-130b
miR-3173-5p
miR-550a-3-5p
miR-152
miR-16-2*
miR-548e
miR-548h-3p
miR-3689a-5p
miR-200b
miR-30a*
miR-874
miR-550
miR-148a
miR-130a
miR-378d
miR-378i
miR-378c
miR-422a
miR-378b
miR-422b
miR-34a
miR-449
miR-193
miR-196b
miR-199b
miR-211
miR-708
let-7e*
miR-362-3p
miR-196a
miR-199a
miR-204
miR-2116*
miR-28
let-7d*
miR-329
miR-449b
miR-641
miR-500b
miR-376b
miR-10b
miR-33b
miR-449c
miR-34c
miR-3617
miR-362
miR-365
miR-376a
miR-10a
miR-611
miR-33
miR-34b
miR-128a
miR-527
miR-212
miR-141
miR-154*
miR-5582-3p
miR-132
miR-133a
miR-548ar-3p
miR-487
