# Supplementary material for: Unique circulating miRNAome signatures captured with distinct molecular states in Lynch syndrome and sporadic colorectal cancer
Source: Mol Oncol. 2026 Oct 7:10.1002/1878-0261.70338. Online ahead of print. doi: 10.1002/1878-0261.70338 (PMC13643886; doi:10.1002/1878-0261.70338)
Supplement: Supplementary file 1 [file MOL2-9999-0-s001.zip › Figure_S2.pdf]

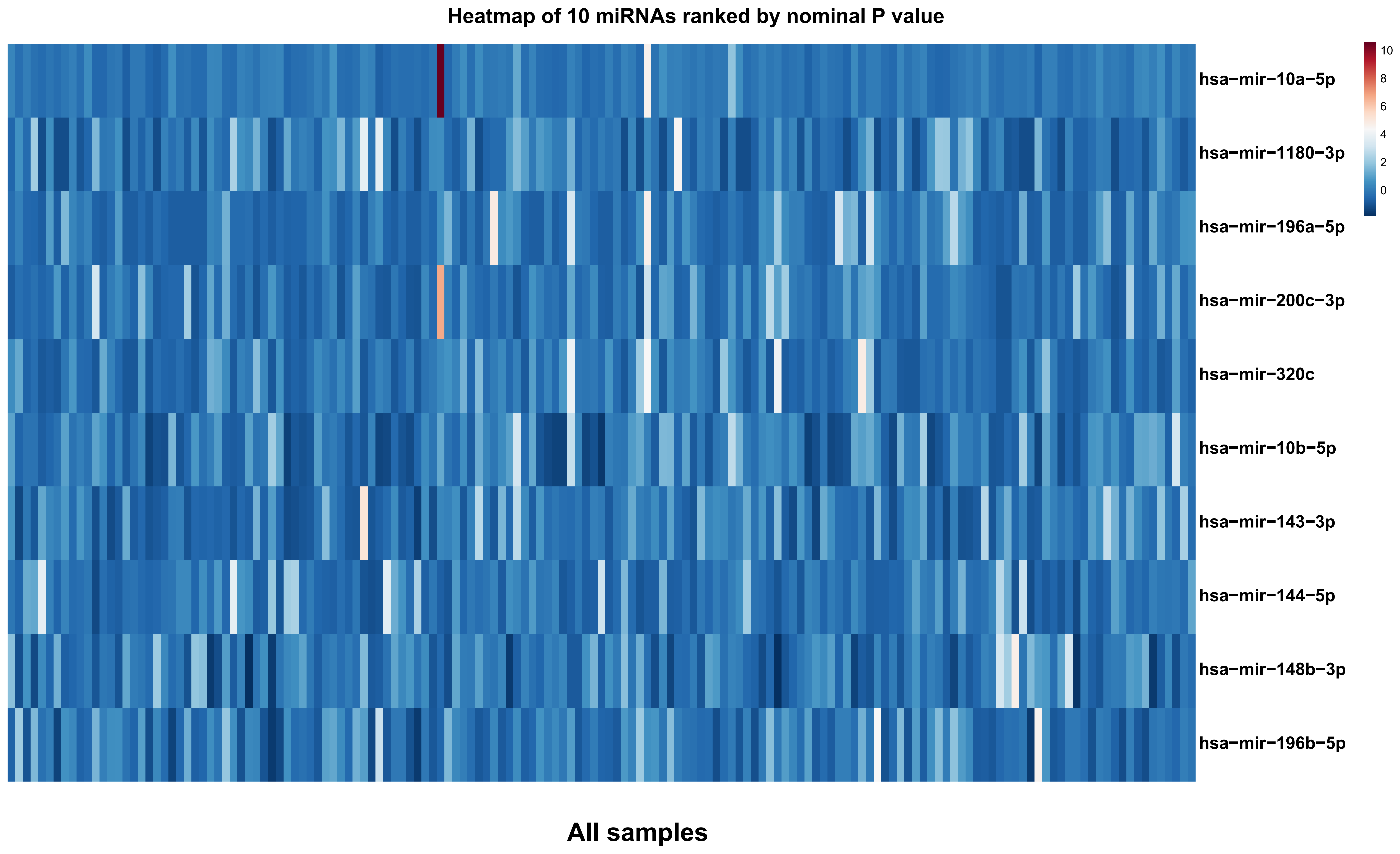
Heatmap of 10 miRNAs ranked by nominal P value
hsa−mir−10a−5p
hsa−mir−1180−3p
hsa−mir−196a−5p
hsa−mir−200c−3p
hsa−mir−320c
hsa−mir−10b−5p
hsa−mir−143−3p
hsa−mir−144−5p
hsa−mir−148b−3p
hsa−mir−196b−5p
10
8
6
4
2
0
All samples
