# Supplementary material for: Unique circulating miRNAome signatures captured with distinct molecular states in Lynch syndrome and sporadic colorectal cancer
Source: Mol Oncol. 2026 Oct 7:10.1002/1878-0261.70338. Online ahead of print. doi: 10.1002/1878-0261.70338 (PMC13643886; doi:10.1002/1878-0261.70338)
Supplement: Supplementary file 1 [file MOL2-9999-0-s001.zip › Figure_S3.pdf]

Age <= 50

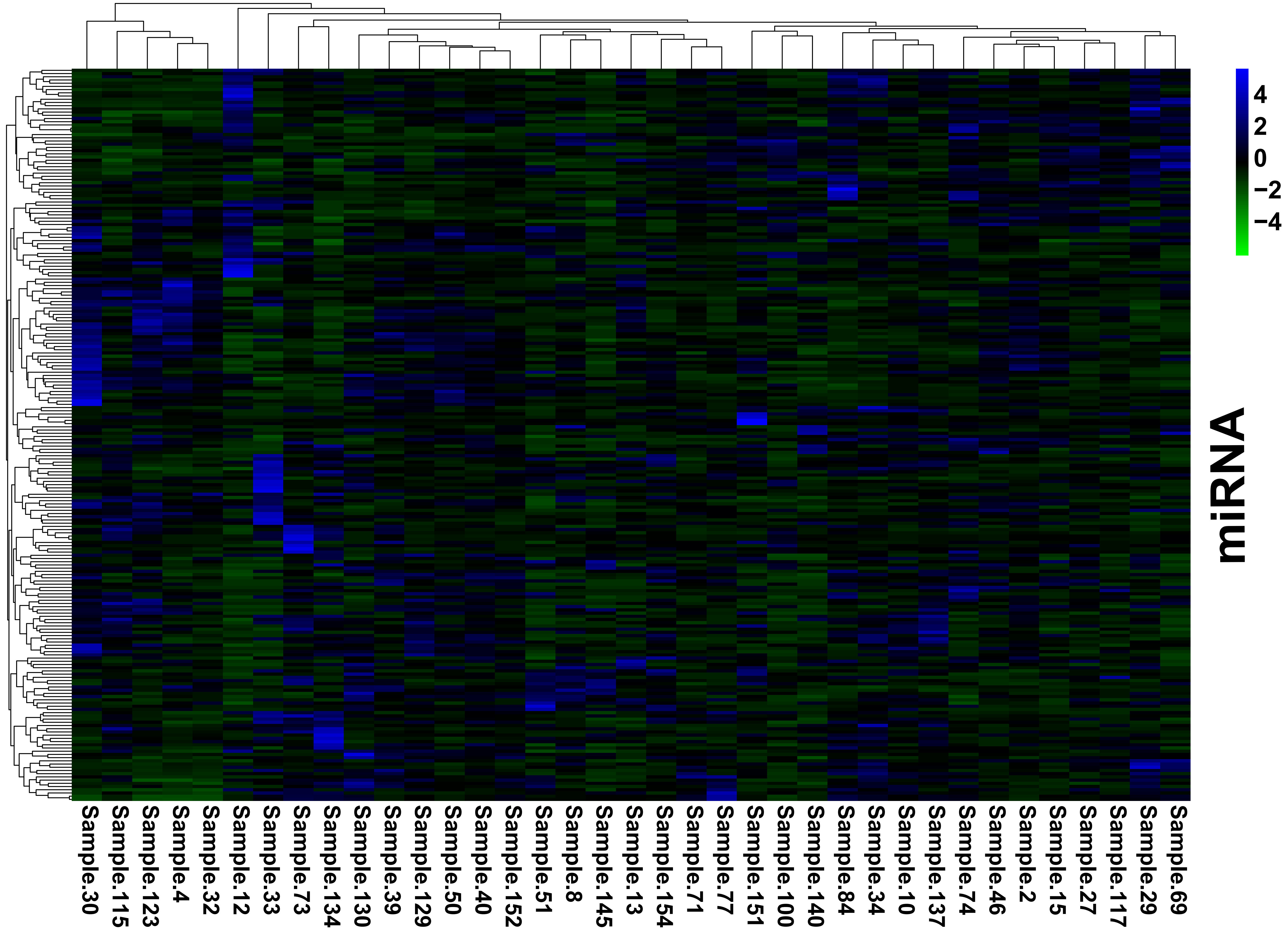

4
2
0
−2
−4
miRNA
Sample.30
Sample.115
Sample.123
Sample.4
Sample.32
Sample.12
Sample.33
Sample.73
Sample.134
Sample.130
Sample.39
Sample.129
Sample.50
Sample.40
Sample.152
Sample.51
Sample.8
Sample.145
Sample.13
Sample.154
Sample.71
Sample.77
Sample.151
Sample.100
Sample.140
Sample.84
Sample.34
Sample.10
Sample.137
Sample.74
Sample.46
Sample.2
Sample.15
Sample.27
Sample.117
Sample.29
Sample.69
