# Supplementary material for: Unique circulating miRNAome signatures captured with distinct molecular states in Lynch syndrome and sporadic colorectal cancer
Source: Mol Oncol. 2026 Oct 7:10.1002/1878-0261.70338. Online ahead of print. doi: 10.1002/1878-0261.70338 (PMC13643886; doi:10.1002/1878-0261.70338)
Supplement: Supplementary file 1 [file MOL2-9999-0-s001.zip › Figure_S6.pdf]

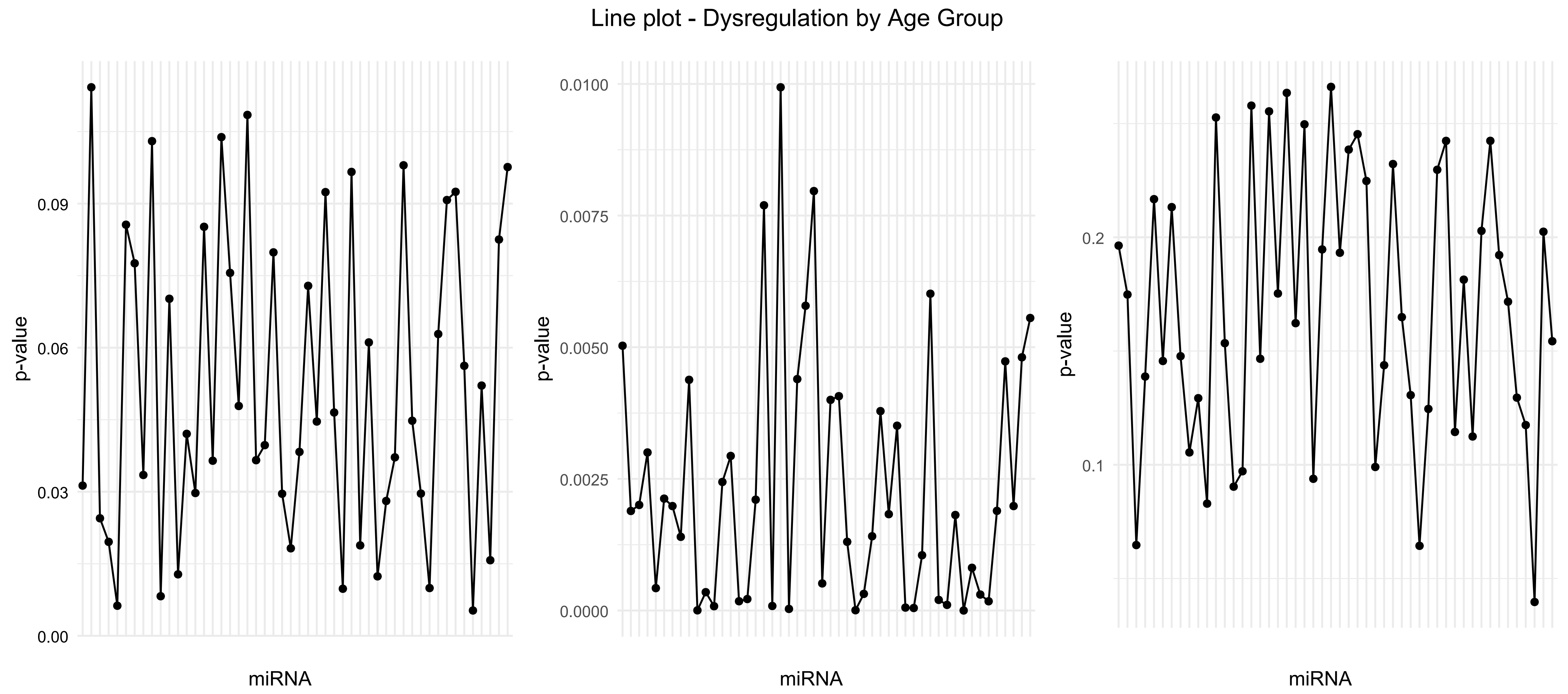
Line plot - Dysregulation by Age Group
p-value
0.00
0.03
0.06
0.09
miRNA
p-value
0.0000
0.0025
0.0050
0.0075
0.0100
miRNA
p-value
0.1
0.2
miRNA
