# Supplementary material for: Unique circulating miRNAome signatures captured with distinct molecular states in Lynch syndrome and sporadic colorectal cancer
Source: Mol Oncol. 2026 Oct 7:10.1002/1878-0261.70338. Online ahead of print. doi: 10.1002/1878-0261.70338 (PMC13643886; doi:10.1002/1878-0261.70338)
Supplement: Supplementary file 1 [file MOL2-9999-0-s001.zip › Figure_S7.pdf]

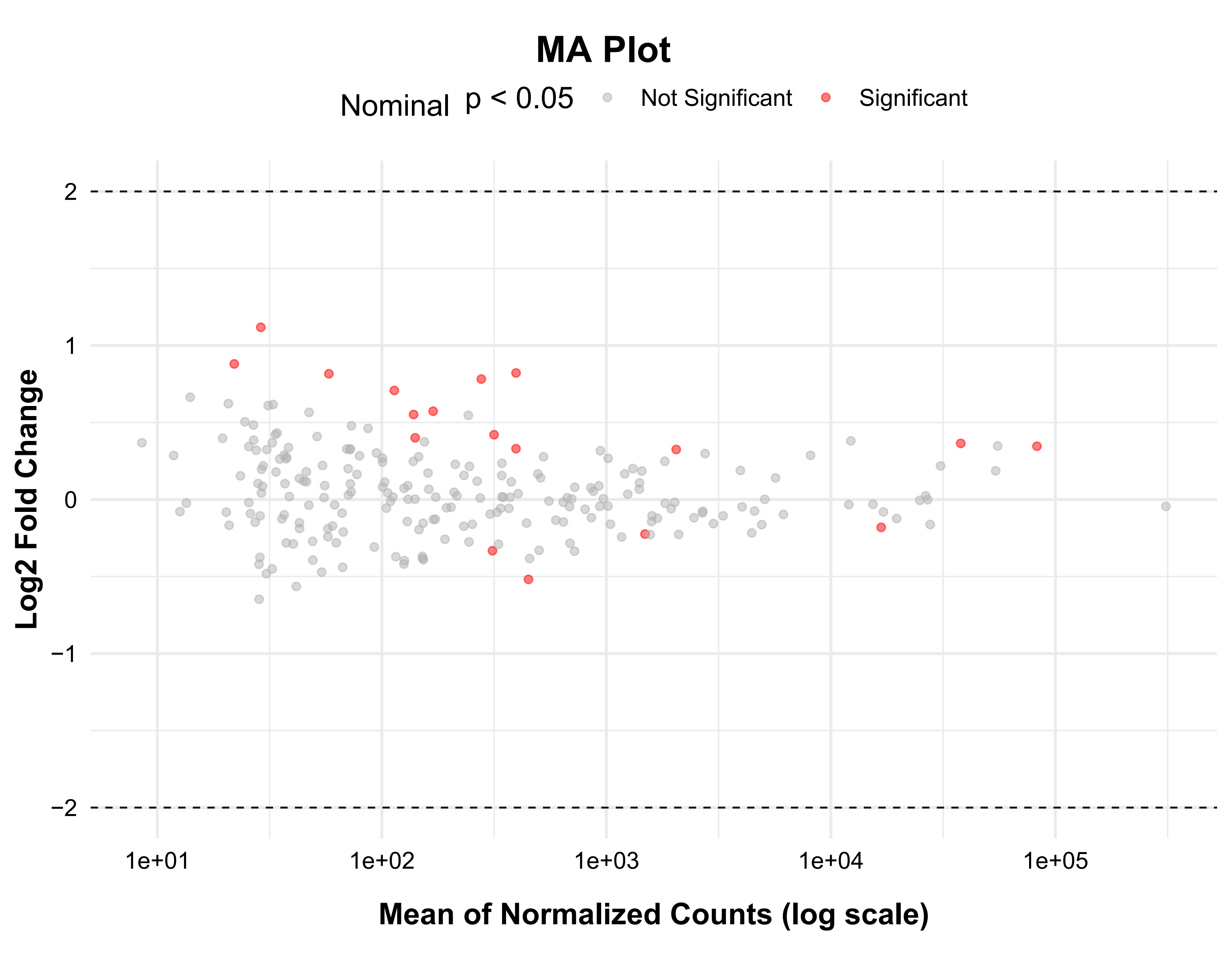

MA Plot
Nominal p < 0.05
Not Significant
Significant
Log2 Fold Change
2
1
0
−1
−2
1e+01
1e+02
1e+03
1e+04
1e+05
Mean of Normalized Counts (log scale)
