# Supplementary material for: Unique circulating miRNAome signatures captured with distinct molecular states in Lynch syndrome and sporadic colorectal cancer
Source: Mol Oncol. 2026 Oct 7:10.1002/1878-0261.70338. Online ahead of print. doi: 10.1002/1878-0261.70338 (PMC13643886; doi:10.1002/1878-0261.70338)
Supplement: Supplementary file 1 [file MOL2-9999-0-s001.zip › Figure_S8.pdf]

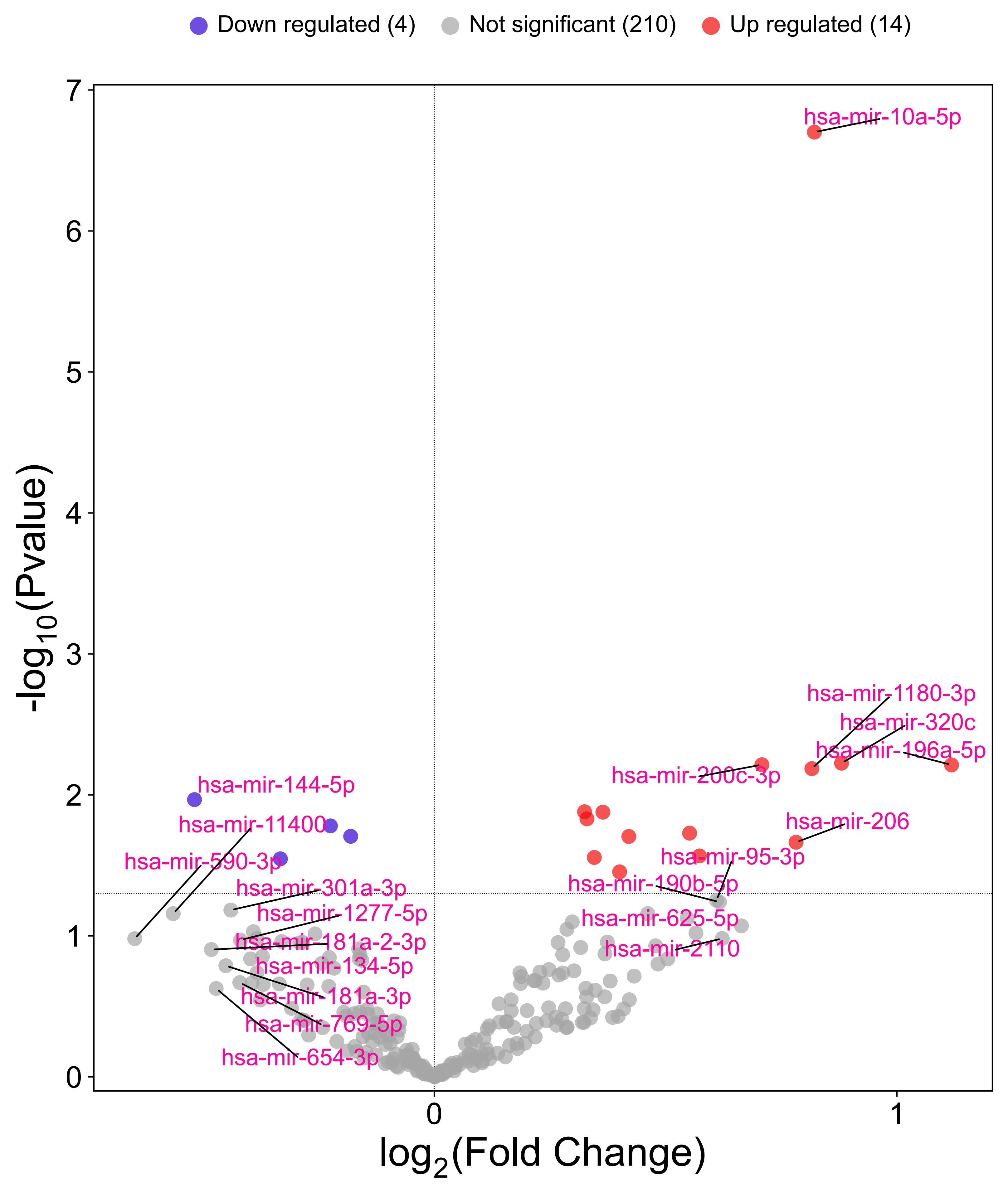

Down regulated (4)
Not significant (210)
Up regulated (14)
-log10(Pvalue)
log2(Fold Change)
7
6
5
4
3
2
1
0
0
1
hsa-mir-10a-5p
hsa-mir-1180-3p
hsa-mir-320c
hsa-mir-196a-5p
hsa-mir-200c-3p
hsa-mir-206
hsa-mir-95-3p
hsa-mir-190b-5p
hsa-mir-625-5p
hsa-mir-2110
hsa-mir-144-5p
hsa-mir-11400
hsa-mir-590-3p
hsa-mir-301a-3p
hsa-mir-1277-5p
hsa-mir-181a-2-3p
hsa-mir-134-5p
hsa-mir-181a-3p
hsa-mir-769-5p
hsa-mir-654-3p
