# Supplementary material for: Unique circulating miRNAome signatures captured with distinct molecular states in Lynch syndrome and sporadic colorectal cancer
Source: Mol Oncol. 2026 Oct 7:10.1002/1878-0261.70338. Online ahead of print. doi: 10.1002/1878-0261.70338 (PMC13643886; doi:10.1002/1878-0261.70338)
Supplement: Supplementary file 1 [file MOL2-9999-0-s001.zip › Figure_S9.pdf]

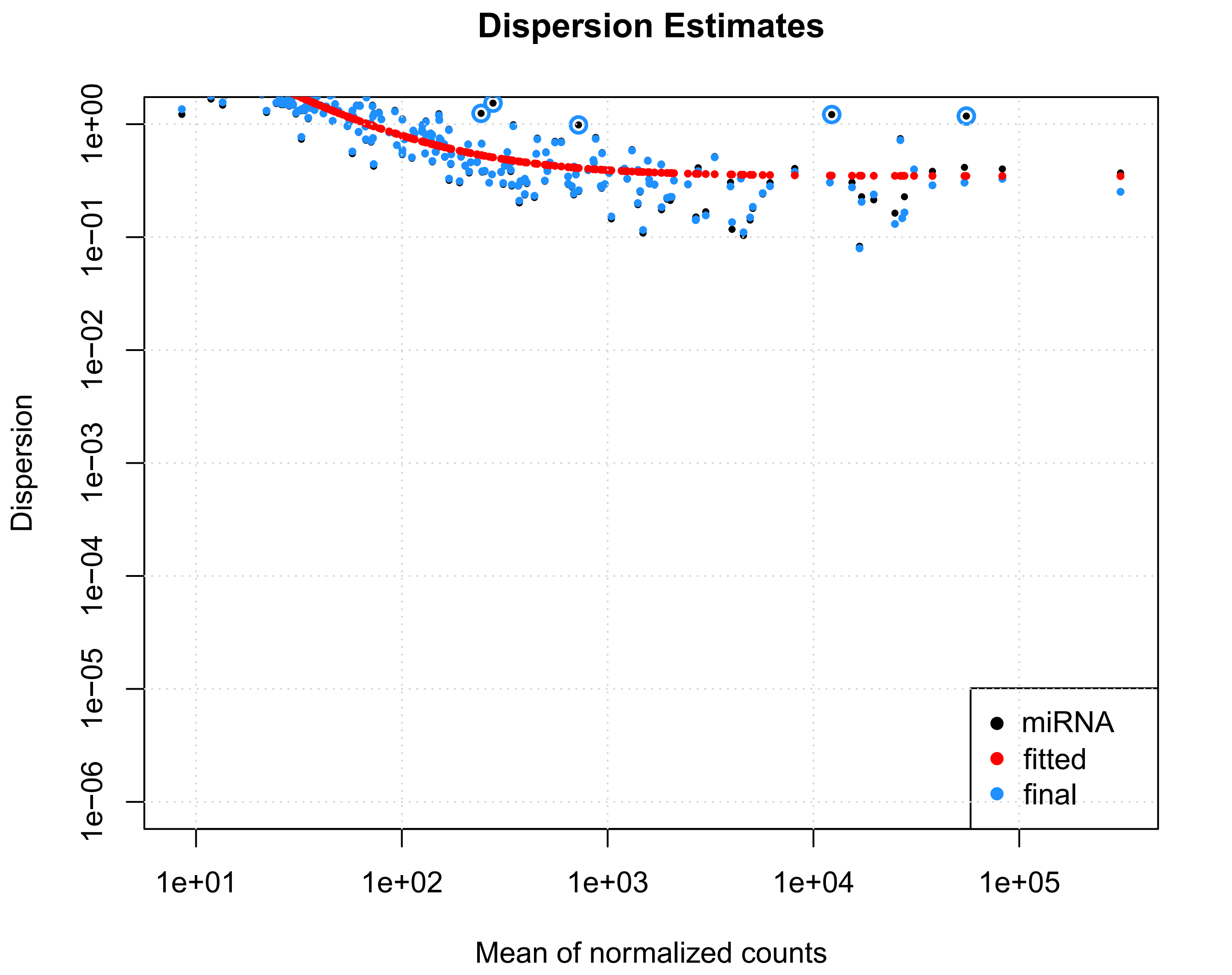

Dispersion Estimates
1e+00
1e−01
1e−02
1e−03
1e−04
1e−05
1e−06
Dispersion
1e+01
1e+02
1e+03
1e+04
1e+05
Mean of normalized counts
miRNA
fitted
final
