# Supplementary material for: Unique circulating miRNAome signatures captured with distinct molecular states in Lynch syndrome and sporadic colorectal cancer
Source: Mol Oncol. 2026 Oct 7:10.1002/1878-0261.70338. Online ahead of print. doi: 10.1002/1878-0261.70338 (PMC13643886; doi:10.1002/1878-0261.70338)
Supplement: Supplementary file 1 [file MOL2-9999-0-s001.zip › Figure_S10.pdf]

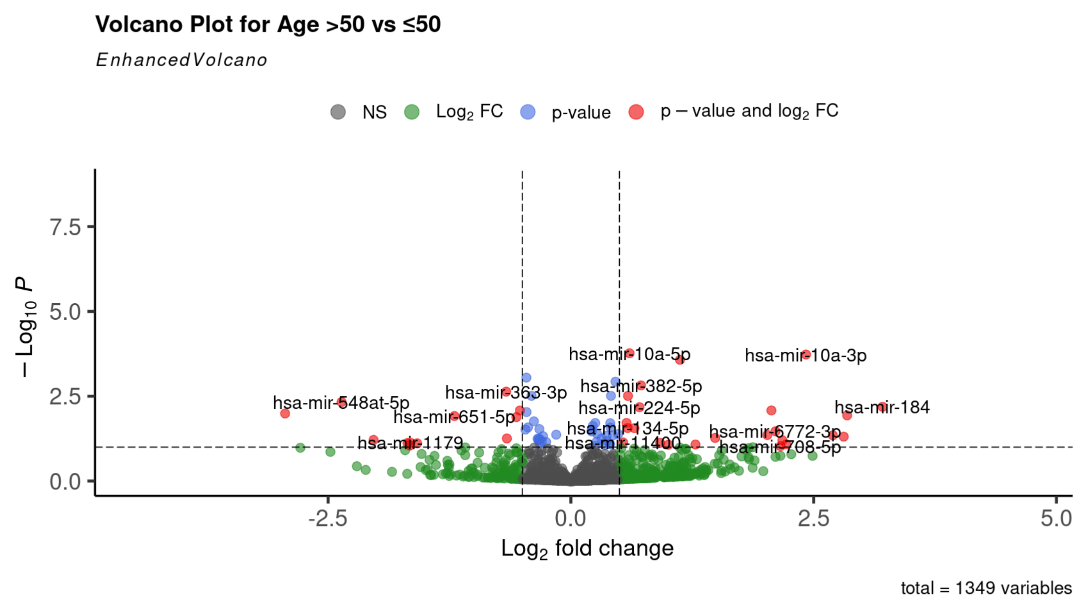

Volcano Plot for Age >50 vs ≤50
EnhancedVolcano
NS
Log_2 FC
p-value
p – value and log_2 FC
$-Log_{10}$ P
7.5
5.0
2.5
0.0
-2.5
0.0
2.5
5.0
Log_2 fold change
hsa-mir-10a-5p
hsa-mir-10a-3p
hsa-mir-382-5p
hsa-mir-363-3p
hsa-mir-548at-5p
hsa-mir-224-5p
hsa-mir-184
hsa-mir-651-5p
hsa-mir-134-5p
hsa-mir-6772-3p
hsa-mir-1179
hsa-mir-11400
hsa-mir-708-5p
total = 1349 variables
