# Supplementary material for: Unique circulating miRNAome signatures captured with distinct molecular states in Lynch syndrome and sporadic colorectal cancer
Source: Mol Oncol. 2026 Oct 7:10.1002/1878-0261.70338. Online ahead of print. doi: 10.1002/1878-0261.70338 (PMC13643886; doi:10.1002/1878-0261.70338)
Supplement: Supplementary file 1 [file MOL2-9999-0-s001.zip › Figure_S11.pdf]

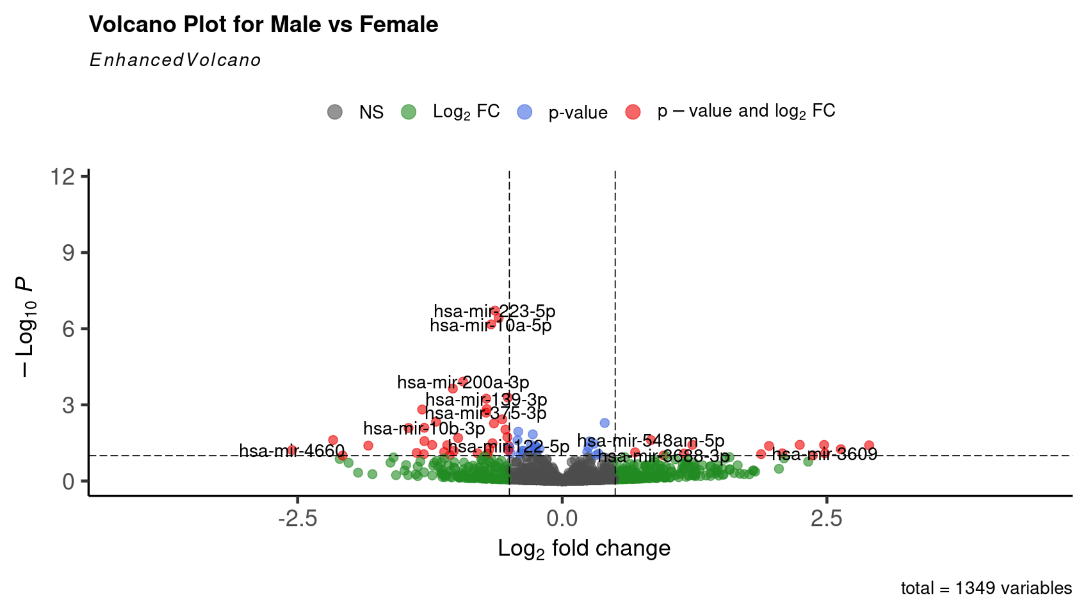

Volcano Plot for Male vs Female
EnhancedVolcano
NS
Log2 FC
p-value
p – value and log2 FC
12
9
6
3
0
– Log10 P
hsa-mir-223-5p
hsa-mir-10a-5p
hsa-mir-200a-3p
hsa-mir-139-3p
hsa-mir-375-3p
hsa-mir-10b-3p
hsa-mir-4660
hsa-mir-122-5p
hsa-mir-548am-5p
hsa-mir-3688-3p
hsa-mir-3609
-2.5
0.0
2.5
Log2 fold change
total = 1349 variables
