# Supplementary material for: Unique circulating miRNAome signatures captured with distinct molecular states in Lynch syndrome and sporadic colorectal cancer
Source: Mol Oncol. 2026 Oct 7:10.1002/1878-0261.70338. Online ahead of print. doi: 10.1002/1878-0261.70338 (PMC13643886; doi:10.1002/1878-0261.70338)
Supplement: Supplementary file 1 [file MOL2-9999-0-s001.zip › Figure_S12.pdf]

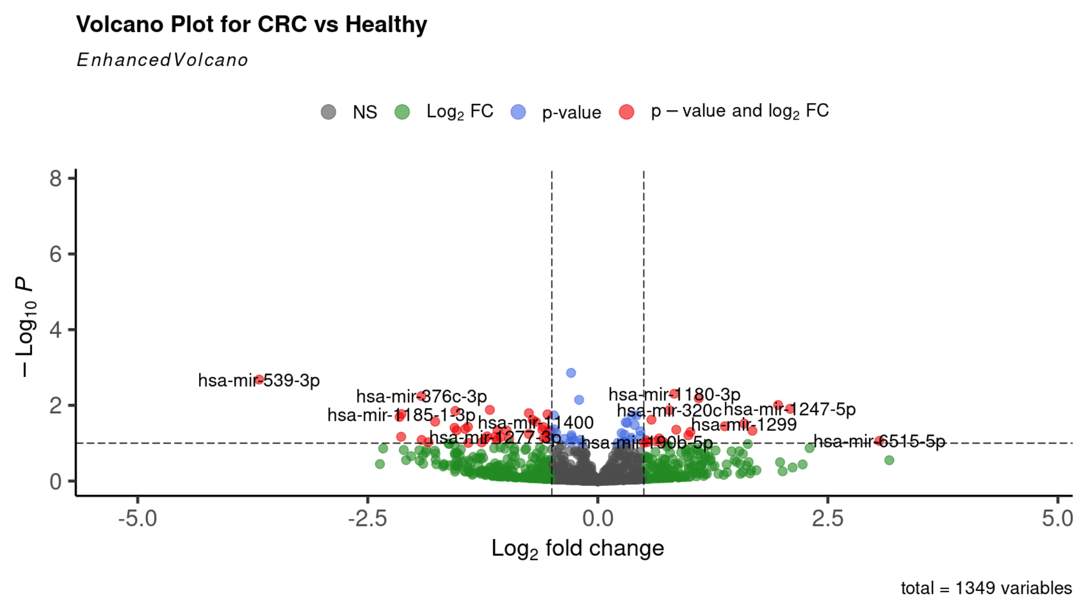
Volcano Plot for CRC vs Healthy
EnhancedVolcano
NS
Log2 FC
p-value
p – value and log2 FC
hsa-mir-539-3p
hsa-mir-376c-3p
hsa-mir-1185-1-3p
hsa-mir-1400
hsa-mir-1277-3p
hsa-mir-1180-3p
hsa-mir-320c
hsa-mir-1247-5p
hsa-mir-1299
hsa-mir-190b-5p
hsa-mir-6515-5p
– Log10 P
0
2
4
6
8
-5.0
-2.5
0.0
2.5
5.0
Log2 fold change
total = 1349 variables
